# A study of the types and manifestations of physicians' unintended behaviors in the DRG payment system

**DOI:** 10.3389/fpubh.2023.1141981

**Published:** 2023-06-27

**Authors:** Wei-Fu Chang, Xin-Yu Yan, Hao Ling, Ting Liu, Ai-Jing Luo

**Affiliations:** ^1^Department of Medical Administration, The Third Xiangya Hospital of Central South University, Changsha, China; ^2^Xiangya School of Public Health, Central South University, Changsha, China; ^3^Key Laboratory of Medical Information Research (Central South University), College of Hunan Province, Changsha, China; ^4^School of Life Sciences, Central South University, Changsha, China; ^5^School of International Pharmaceutical Business, China Pharmaceutical University, Nanjing, China; ^6^Information Network Center, The Second Xiangya Hospital of Central South University, Changsha, China; ^7^Clinical Research Center for Cardiovascular Intelligent Healthcare in Hunan Province, Changsha, China

**Keywords:** DRG payment, unintended behaviors, physicians, interrupted time series analysis, negotiation mechanism

## Abstract

**Introduction:**

In recent years, China has implemented the Diagnosis Related Groups (DRG) payment system as part of its healthcare insurance reimbursement policy. Numerous studies have focused on the effectiveness of DRG payment system in controlling unreasonable growth in medical expenses. However, there has been no systematic report on the types of unintended behaviors exhibited by doctors under the DRG payment system.

**Methods:**

The study first utilized interrupted time series analysis to analyze medical records and insurance data from eight hospitals. It investigated the data changes in MDC and ADRG groups before and after the implementation of the DRG payment system. Subsequently, a semi-structured interview method was employed to conduct qualitative research on the unintended behaviors of physicians, aiming to gain a more accurate understanding of specific changes in physician behavior after the implementation of the DRG payment system.

**Results:**

This study discovered that doctors engage in unintended behaviors within the framework of the DRG payment system.

**Discussion:**

In the early implementation of the DRG payment system in China, the contradictions between the flawed DRG payment methods and supporting systems and the actual diagnostic and treatment work manifested in the form of unintended doctor behaviors. Most of these unintended behaviors can be considered reasonable feedback from doctors to cope with the existing system flaws. They are conducive to identifying the deficiencies in China's DRG payment system and suggesting directions for improvement.

## 1. Introduction

Since the popularization of healthcare insurance systems worldwide, the issue of soaring medical costs has been a matter of great concern. Initially, healthcare insurance was based on the actual treatment costs incurred by patients. Under such payment systems, patients desired to receive more and better medical services, while doctors aimed to increase their medical service income. The intentions of both parties led to a rapid increase in medical expenses and healthcare fund expenditure. Consequently, countries have implemented policies to redesign the system at the payment level, replacing the payment based on actual costs with fixed payments based on disease types. This shift aims to reduce wasteful spending of healthcare funds and improve their efficiency (1, 2). Diagnosis Related Groups (DRG) is one of the mainstream payment methods based on fixed payments according to disease types.

The DRG payment system accurately calculates the amount of healthcare insurance that should be paid for each case under different conditions, considering factors such as the main diagnosis, major surgical procedures, complications and comorbidities, age, admission status, discharge outcome, and geographic location. Research has shown that the DRG payment system effectively controls healthcare costs within a reasonable range that matches the average treatment costs for each disease type. It improves the utilization efficiency of healthcare funds and helps control the rise in medical expenses (3–5). However, at the same time, doctors, who are in a position of informational advantage, may engage in unintended behaviors under the DRG payment system. Examples of such unintended behaviors include early discharge (discharging patients before complete recovery but at a low risk) and cherry-picking (6) (preferring cases that result in higher surplus after deducting healthcare costs from the insurance payment). These unintended behaviors, occurring within the framework of the DRG payment system, result in negative consequences such as decreased effectiveness of diagnosis and treatment services and wastage of healthcare funds.

Different from countries that were early adopters of DRG payment, China has its own distinct cultural background and healthcare system characteristics. Firstly, as a developing country, China had a relatively slow development of its medical system and healthcare payment system. When DRG payment system was widely implemented in Western countries at the end of the last century, China was still in the process of establishing a universal healthcare insurance system from scratch. The formal implementation of the DRG payment system in China began only in recent years. It was not until 2014 that Beijing first officially applied DRG grouping for performance evaluation, and in 2019, the National Medical Insurance Administration conducted pilot reforms of the DRG payment system in 30 cities nationwide, marking a large-scale promotion of the DRG payment system in China. Secondly, China's social management is characterized by a high level of government authority and responsibility. Compared to Western countries, the government has a greater scope of management and assumes more social responsibilities.

Based on previous studies on the DRG payment system, unintended behaviors are commonly observed (7–17). We assume that China has also experienced unintended behaviors after implementing the DRG payment system. However, the specific types of unintended behaviors, their manifestations, and characteristics in clinical practice require urgent research.

### 1.1. Chinese healthcare insurance fund supervision system

The healthcare insurance system in China is centered around the administrative unit of the Medical Security Bureau, which was established in 2018 at the national level. It has subsidiary institutions at the provincial, municipal, and county levels responsible for formulating regulations, policies, plans, and standards for healthcare insurance and medical assistance systems. The National Medical Security Bureau also oversees the organization and implementation of healthcare insurance-related affairs. It includes a Fund Supervision Division, which is responsible for regulating medical service behaviors and medical expenses covered by the insurance. The supervision is mainly carried out through manual and on-site inspections, sometimes involving third-party professional organizations. Manual inspections involve accounting audits of medical expense data by the Fund Supervision Division to evaluate the utilization of healthcare funds at a macro level. On-site inspections are conducted without prior notice and have been effective in identifying violations. In 2020 alone, the National Medical Security Bureau organized 61 on-site inspections, uncovering a total of 540 million yuan in illegal or irregular funds.

Local healthcare insurance agencies, which are affiliated with the Ministry of Human Resources and Social Security, are not directly governed by the Medical Security Bureau in terms of administrative structure. However, they are managed by the Medical Security Bureau in terms of operations. These agencies perform functions such as agreement management and auditing of healthcare fee settlement payments. Agreement management involves the signing of designated agreements between healthcare insurance agencies and medical institutions or retail pharmacies. It has two stages: the first stage is admission supervision, which involves evaluating the eligibility of medical and pharmaceutical institutions based on certain threshold criteria, such as the number of physicians, pharmacists, and beds. The second stage of agreement management involves the primary healthcare insurance agency overseeing the compliance of the party to the agreement with its terms and conditions. The basic contents of the service agreement include provisions on the target population, service scope, service content, service quality, fee settlement, and breach of contract handling.

The public, businesses, associations, and the media obtain information through their daily activities and participate in social supervision of healthcare institutions. They can also provide policy suggestions to the Medical Security Bureau. Prior to the introduction of new policies, the Medical Security Bureau seeks public opinions through online and offline channels and specifically reaches out to policy stakeholders such as industry associations and businesses for feedback. This communication channel remains open during the policy implementation process.

**Figure 1:**
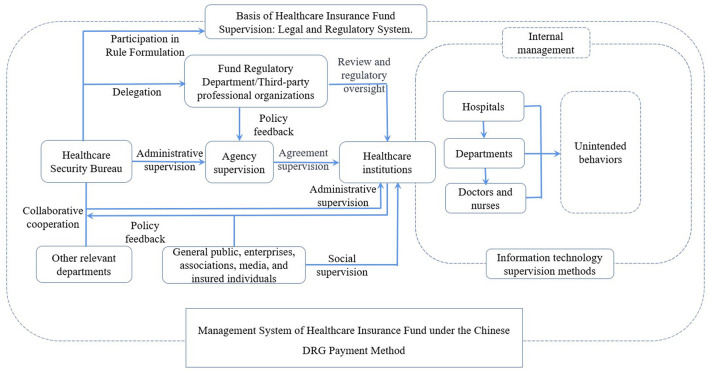
Departments involved in the supervision of China's healthcare insurance fund and their main responsibilities.

The supervision of the Chinese healthcare insurance fund is primarily carried out by the government through the Medical Security Bureau. However, certain aspects of fund supervision involve joint inspections and case referrals with the involvement of multiple government departments. The Medical Security Bureau takes the lead in cooperation, while other departments such as health authorities, drug regulatory agencies, financial departments, audit institutions, and public security agencies provide auxiliary support and fulfill their respective responsibilities, rights, and obligations. The division of labor among these departments can be seen in Figure 1.

In China, public hospitals are the main type of healthcare institutions, while private hospitals have a relatively lower proportion. Healthcare institutions maintain close contact with the Medical Security Bureau and participate in consultations on healthcare insurance-related policies and their implementation. Due to its administrative authority, the Medical Security Bureau holds a relatively stronger position in these processes. Healthcare institutions utilize information technology within their organizations and rely on a vertical organizational structure to monitor unexpected behaviors.

### 1.2. Introduction to CHS-DRG grouping

In 2011, China conducted its first pilot project on the DRG payment system in Beijing, using the BJ-DRG developed by the Beijing Medical Insurance Association. Subsequently, China introduced other regional DRG systems such as CN-DRG jointly developed by the National Health Commission's Health Administration and Medical Administration Bureau and the Beijing Municipal Health Commission Information Center, CR-DRG developed by the Grassroots Health Bureau of the National Health Commission, and C-DRG developed by the National Health Commission. These are all successful cases of DRG application in China. Building on the successful experiences of these DRG applications, the National Medical Security Bureau officially released the “National Medical Security Diagnosis Related Group (CHS-DRG) Grouping Scheme” in October 2019, marking the formation of China's national-level CHS-DRG system. In 2020, the National Medical Security Bureau led negotiations and initiated CHS-DRG payment pilot projects in 30 cities (18, 19).

DRG grouping utilizes diagnostic and treatment information from the patient's hospital admission record to categorize cases into a Major Diagnostic Category (MDC). Then, based on the primary treatment approach, cases are further divided into Adjacent Diagnosis Related Groups (ADRG) that are clinically relevant. Additionally, factors such as age, presence of complications or comorbidities (CC), and presence of major complications or comorbidities (MCC) are taken into account. Following the principles of clinical consistency and similar resource consumption, cases are finally assigned to different DRG groups. Please refer to Appendices A, B for MDC grouping and ADRG primary grouping.

The CHS-DRG grouping scheme stipulates that the 26 major diagnostic categories (MDCs) and 376 core disease diagnosis-related groups (ADRGs) should be consistent across all pilot cities. However, due to differences in socioeconomic levels and city sizes among the pilot cities, significant variations exist in data reporting standards, data quality, healthcare expenditure levels, and residents' health conditions. Therefore, pilot cities need to consider factors such as city size, regional differences, and disease profiles to select appropriate statistical methods for developing complication and comorbidity tables, which form the basis for the DRG grouping. As the focus of this study is not on DRG grouping techniques, the specific differences in DRG grouping and complications and comorbidities across regions are not detailed here.

Finally, due to the relevance of DRG codes to the study results, a brief introduction to DRG codes is provided. A DRG code consists of four characters, comprising uppercase English letters A–Z and Arabic numerals 0–9. The specific meanings of each DRG code are as follows: the first character is an uppercase English letter, with A–Z representing the 26 major diagnostic categories (MDCs). The second character is an uppercase English letter representing the type of DRG: A, B, C, D, E, F, G, H, J represent surgical sections; K, L, M, N, P, Q represent non-operative sections; R, S, T, U, V, W, X, Y, Z represent medical sections. The third character is an Arabic numeral 1–9, indicating the sequential code for the DRG. The fourth character is an Arabic numeral representing the presence of complications or comorbidities. “1” indicates the presence of major complications or comorbidities, “3” indicates the presence of general complications or comorbidities, “5” indicates the absence of complications or comorbidities, and “9” indicates an unspecified situation. The relative weight (RW) of a DRG represents the weight assigned to each DRG based on its resource consumption level, reflecting the relative resource consumption of that DRG compared to other diseases. An RW value of 1 for a DRG indicates that the healthcare fund consumption for that particular group is equal to the average for all diseases.

## 2. Materials and methods

### 2.1. Study subject

The study selected a pilot DRG payment system city in China, where there are 32 hospitals at the secondary level and above by the end of 2020. Of the 32 hospitals, 8 ones participated in the first DRG payment system pilot program launched in 2019. And these 8 hospitals were selected for the study.

### 2.2. Data

The data consisted of medical records and insurance settlement data of inpatients covered by basic medical insurance from eight hospitals in the DRG pilot city in Hunan Province, from January 1, 2021, to September 30, 2021, totaling 188,256 cases. After obtaining the data, preprocessing was conducted to address missing or inaccurate data fields and to ensure the linkage between medical record data and insurance settlement data. The final analyzed dataset included 169,778 cases, accounting for 90.18% of the total data. The specific data types included in the medical record and insurance settlement data were: admission date, discharge date, length of hospital stay, primary diagnosis code at discharge, secondary diagnosis codes at discharge, primary procedure code, secondary procedure codes, whether there was a readmission plan within 31 days of discharge, DRG grouping, DRG grouping weight, total medical expenses, total hospitalization expenses, basic medical insurance reimbursement amount, and patient's out-of-pocket expenses (see Table 1). The provided data were obtained from the Hunan Provincial Medical Insurance Bureau, and they underwent de-identification and ethical review procedures.

**Table 1:** Medical record admission data and medical insurance settlement data.

**Variables**	**Variable assignment**	**Type of variable**
Gender	1 = male; 2 = female.	Nominal variables
Age	—	Interval variables
Hospital name	1 = A; 2 = B; 3 = C; 4 = D; 5 = E; 6 = F; 7 = G; 8 = H.	Nominal variables
Admission date	—	Ordinal variables
Discharge date	—	Ordinal variables
Principal diagnosis (Primary)	—	Nominal variables
Disease code (primary)	—	Nominal variables
Principal procedure	—	Ordinal variables
Procedure code	—	Ordinal variables
Secondary procedure	—	Nominal variables
Procedure code	—	Nominal variables
Planned readmission	1 = yes; 2 = no.	Nominal variables
Total cost	—	Ratio variables
Out-of-pocket amount	—	Ratio variables
Medical insurance fund payment amount	—	Ratio variables
DRG grouping	—	Nominal variables
DRG group weight	—	Ratio variables
Number of other diagnoses	—	Ratio variables

### 2.3. Study method

The study first utilized interrupted time series analysis, a quantitative method, to analyze medical records and insurance data from eight hospitals. It investigated the data changes in MDC and ADRG groups before and after the implementation of the DRG payment system and discussed potential unintended behaviors that may exist. Subsequently, a semi-structured interview method was employed to conduct qualitative research on the unintended behaviors of physicians, aiming to gain a more accurate understanding of specific changes in physician behavior after the implementation of the DRG payment system. This qualitative study provided further evidence for the quantitative research and contributed to obtaining more credible research results.

#### 2.3.1. Interrupted time series analysis

To identify the indicator changes before and after the implementation of the DRG payment system, the study used interrupted time series (ITS) analysis, a statistical method employing segmented regression models. The implementation of the DRG payment system was considered as the intervention point, and a dummy variable was used to mark the data before and after the intervention. Multivariate segmented regression analyses were performed on the indicator data during the pre- and post-intervention periods to estimate the level and trend changes of the indicators. Interrupted time series research methods can control for baseline levels and trends and address autocorrelation issues using the generalized difference-in-differences approach. This approach allows for the accurate evaluation of the effects of the intervention and the relationship between the dummy variable and the independent variable in the study (20, 21).

Using the collected data from eight hospitals before and after the implementation of the DRG payment system, with the implementation of the DRG payment system as the intervention point and the time of inclusion as the independent variable, various healthcare service indicators were used as dependent variables. Linear regression equations were constructed for different time segments, and the model formula is as follows:


$$\begin{array}{l} Y_{t}\;=\;\beta_{0}\;+\;\beta_{1}\;\times \;time\;+\;\beta_{2}\;\times \;DRG\;+\;\beta_{3}\;\times \;time_{DRG}\;+\;e_{t} \end{array}$$

The significance of each parameter in the equation is as follows:

*Y*_*t*_ is the indicator of each dependent variable, and this study includes indicators such as the proportion of cases in the DRG with severe comorbidities or complications, the number and proportion of cases with RW <1, and the unscheduled hospital readmission rate within 31 days. β_0_ is the baseline value, which is an estimate of the level of the dependent variable before DRG implementation; β_1_ is the baseline slope estimate, which is an estimate of the trend of the dependent variable over time t before DRG implementation; *time* is a continuous variable of time, with one observation period per half month (18 data time scales in total); β_2_ the level change estimate, i.e., the change in the value of the dependent variable at the instant of DRG implementation, indicating the instantaneous effect of DRG implementation; β_3_ is the trend change estimate, which is the difference between the trend value of the dependent variable after the implementation of the DRG and the trend value before the implementation, and represents the change in the trend of the dependent variable change after the implementation of the DRG; *time*_*DRG*_ is assigned to 0 before the policy implementation and 1–8 after the policy implementation; *e*_*t*_ is the error term.

The change in indicators before and after the implementation of the DRG payment system was analyzed using the interrupted time series segmentation model in SPSS 23.0 statistical software, with a test level of 0.05 and a two-sided test.

#### 2.3.2. Semi-structured interviews

In this study, three hospitals from a city participating in the national pilot program for the DRG payment system were selected as research sites. Semi-structured interviews were conducted with physicians, nurses, and hospital administrators in selected departments using a sample size following the principle of data saturation. The interviews commenced in early March 2022 and concluded by the end of April 2022, involving 36 selected participants. The interview content was pre-designed based on a progressive relationship and included the following topics:

1. Implementation of the DRG payment system in the hospital or department.
2. Effects of the DRG payment system on physician behavior.
3. Types of unintended behaviors observed after the implementation of the DRG payment system.

During the interviews, a neutral stance was maintained, and the final results were based on the interviewees' own experiences and viewpoints, avoiding any influence or bias toward the opinions of the interviewed parties or the supervising healthcare authorities. The privacy of the interviewees was protected, and their identities were anonymized due to the potentially controversial nature of describing and evaluating unexpected physician behaviors under the DRG payment system, as some views may not be widely accepted and public disclosure could have adverse consequences for the interviewees.

## 3. Result

### 3.1. Descriptive statistical analysis results

#### 3.1.1. Analysis of the number and percentage of cases in each DRG

During the 5 months before the implementation of the DRG payment system, the total number of cases in the eight hospitals was 97,192, which were grouped into 393 DRGs. In the 4 months after the reform of the DRG payment system, the total number of cases was 72,589, which were grouped into 391 DRGs. After excluding DRG groups with a small number of cases, the top 50 DRG groups in terms of case count after the implementation of the DRG payment system are listed, totaling 98,843 cases, accounting for 58.2% of the total cases. Please refer to Table 2 for details.

**Table 2:** Analysis of the number and proportion of cases in each DRG before and after the implementation of the DRG payment system.

**DRG**	**RW**	**Total**		**Before implementation**		**After implementation**		**Percentage increase**
		**Number of cases**	**Proportion**	**Number of cases**	**Proportion**	**Number of cases**	**Proportion**	
BR23	0.8	9,415	5.55%	5,006	5.15%	4,409	6.07%	17.93%
RE19	0.5	4,346	2.56%	773	0.80%	3,573	4.92%	518.92%
FR33	0.9	5,590	3.29%	2,954	3.04%	2,636	3.63%	19.48%
FR19	1.1	4,431	2.61%	2,114	2.18%	2,317	3.19%	46.76%
IU29	0.7	4,489	2.64%	2,511	2.58%	1,978	2.73%	5.48%
ES25	0.4	4,473	2.63%	2,612	2.69%	1,861	2.56%	−4.60%
BR21	1.2	3,418	2.01%	1,862	1.92%	1,556	2.14%	11.89%
ER39	1.3	1,912	1.13%	505	0.52%	1,407	1.94%	273.06%
ES23	0.8	4,549	2.68%	3,186	3.28%	1,363	1.88%	−42.72%
DT15	0.3	2,650	1.56%	1,360	1.40%	1,290	1.78%	27.01%
KS13	0.9	2,141	1.26%	859	0.88%	1,282	1.77%	99.84%
FM35	1.2	2,852	1.68%	1,687	1.74%	1,165	1.60%	−7.53%
ET25	0.8	3,650	2.15%	2,530	2.60%	1,120	1.54%	−40.72%
ET21	1.1	3,483	2.05%	2,458	2.53%	1,025	1.41%	−44.16%
RU19	0.8	2,380	1.40%	1,488	1.53%	892	1.23%	−19.73%
EJ11	2.1	1,343	0.79%	545	0.56%	798	1.10%	96.06%
LK19	2.1	1,167	0.69%	401	0.41%	766	1.06%	155.78%
GW13	0.7	2,417	1.42%	1,685	1.73%	732	1.01%	−41.83%
RW23	0.7	1,834	1.08%	1,125	1.16%	709	0.98%	−15.61%
GW15	0.4	2,189	1.29%	1,482	1.52%	707	0.97%	−36.12%
GF19	1.4	1,520	0.90%	827	0.85%	693	0.95%	12.20%
KS11	1.2	839	0.49%	259	0.27%	580	0.80%	199.85%
GZ13	0.8	1,544	0.91%	964	0.99%	580	0.80%	−19.44%
GV13	0.7	1,382	0.81%	813	0.84%	569	0.78%	−6.29%
BX23	0.8	1,980	1.17%	1,414	1.45%	566	0.78%	−46.40%
BR25	0.6	963	0.57%	412	0.42%	551	0.76%	79.07%
EJ13	1.5	1,043	0.61%	497	0.51%	546	0.75%	47.10%
LL19	1.2	763	0.45%	222	0.23%	541	0.75%	226.30%
EX25	0.3	1,559	0.92%	1,026	1.06%	533	0.73%	−30.44%
RW21	0.9	1,153	0.68%	629	0.65%	524	0.72%	11.55%
IU19	0.7	1,429	0.84%	906	0.93%	523	0.72%	−22.71%
PU15	0.4	973	0.57%	523	0.54%	450	0.62%	15.21%
FM13	6.9	1,237	0.73%	808	0.83%	429	0.59%	−28.91%
FV23	0.7	1,480	0.87%	1,065	1.10%	415	0.57%	−47.82%
LU13	0.5	845	0.50%	431	0.44%	414	0.57%	28.62%
IJ15	1.2	669	0.39%	260	0.27%	409	0.56%	110.63%
NF19	0.7	1,086	0.64%	680	0.70%	406	0.56%	−20.05%
LR15	1	781	0.46%	391	0.40%	390	0.54%	33.56%
SR11	1.4	570	0.34%	189	0.19%	381	0.52%	169.92%
RW25	0.6	918	0.54%	559	0.58%	359	0.49%	−14.01%
GZ15	0.5	862	0.51%	506	0.52%	356	0.49%	−5.79%
FM31	1.5	744	0.44%	405	0.42%	339	0.47%	12.08%
GS13	1	805	0.47%	466	0.48%	339	0.47%	−2.59%
CB19	1.1	813	0.48%	475	0.49%	338	0.47%	−4.72%
NC19	2.1	655	0.39%	323	0.33%	332	0.46%	37.63%
RC19	6.2	634	0.37%	305	0.31%	329	0.45%	44.44%
KS15	0.7	531	0.31%	203	0.21%	328	0.45%	116.35%

From the Table 2, it can be observed that after the implementation of the DRG payment system, there have been significant changes in the case count and proportion in some DRG groups, with 35 out of 50 DRG groups experiencing a change of more than 15%. Some DRG groups have shown a significant increase in case count. For example, in the RE19 group, which corresponds to malignant proliferative disorders without differentiation of complications/comorbidities, receiving chemotherapy and/or other treatments with a weight of 0.5, there were 773 cases before the implementation of the DRG payment system, accounting for 0.80% of all cases. After the implementation of the DRG payment system, there were 3,573 cases, accounting for 4.92%. This represents an increase of 518.92%. Conversely, some DRG groups have experienced a significant decrease in case count. For example, in the FV23 group, corresponding to hypertension with general complications or comorbidities, with a weight of 0.7, there were 1,065 cases before the implementation of the DRG payment system, accounting for 1.10% of all cases. After the implementation of the DRG payment system, there were 415 cases, accounting for 0.57%, representing a decrease of 47.82%. Subsequently, further research can be conducted from the perspective of MDC and ADRG to analyze the changes in case count and investigate the behavioral changes of physicians before and after the implementation of the DRG payment system.

#### 3.1.2. Analysis of the number and proportion of cases in DRG with serious comorbidities or complications

After the implementation of the DRG payment system, changes were observed in both the case count and proportion among DRG groups with different levels of severity for complications or comorbidities. Specifically, the fourth digit of the disease code represents the presence of severe complications or comorbidities (1) and general complications or comorbidities (3). Significant changes (*p*-value <0.05) were identified by selecting ADGR groups with notable differences after the implementation of the DRG payment system. A total of 10 ADGR groups were identified (see Table 3). Taking ADGR group ET1 as an example, which includes two DRG groups (ET11 and ET15), the proportion of cases in ET11 increased from 36.96% before the implementation of the DRG payment system to 63.16% after the implementation, representing a 26.20% increase. Conversely, the proportion of cases in ET15 decreased from 63.04% before the implementation to 36.84% after the implementation, representing a 26.20% decrease. A chi-square test confirmed that these changes were statistically significant (*p*-value = 0.001 <0.05).

**Table 3:** Number and proportion of cases with serious comorbidities and complications in each DRG.

**ADRG**	**DRG**	**Before implementation**		**After implementation**		**Increase**	**χ^2^**	***P***
		**Number of cases**	**Proportion**	**Number of cases**	**Proportion**			
ED1	ED11	151	44.41	115	52.04	7.62	15.891	0.000
	ED13	166	48.82	75	33.94	−14.89		
	ED15	23	6.76	31	14.03	7.26		
ER1	ER11	174	50.88	98	60.49	9.62	4.092	0.043
	ER15	168	49.12	64	39.51	−9.62		
ES1	ES11	194	29.94	141	36.72	6.78	7.004	0.030
	ES13	342	52.78	171	44.53	−8.25		
	ES15	112	17.28	72	18.75	1.47		
ET1	ET11	34	36.96	60	63.16	26.20	12.835	0.000
	ET15	58	63.04	35	36.84	−26.20		
FM3	FM31	405	19.36	339	22.54	3.18	5.394	0.020
	FM35	1,687	80.64	1165	77.46	−3.18		
FW1	FW11	172	37.97	66	47.14	9.17	11.285	0.004
	FW13	257	56.73	59	42.14	−14.59		
	FW15	24	5.30	15	10.71	5.42		
LV1	LV11	175	29.86	148	31.69	1.83	15.759	0.000
	LV13	388	66.21	274	58.67	−7.54		
	LV15	23	3.92	45	9.64	5.71		
QS4	QS41	45	39.47	60	50.85	11.37	6.242	0.044
	QS43	55	48.25	38	32.20	−16.04		
	QS45	14	12.28	20	16.95	4.67		
RW2	RW21	629	27.19	524	32.91	5.72	14.880	0.001
	RW23	1,125	48.64	709	44.54	−4.10		
	RW25	559	24.17	359	22.55	−1.62		
SR1	SR11	189	49.22	381	57.55	8.33	44.936	<0.0001
	SR13	88	22.92	204	30.82	7.90		
	SR15	107	27.86	77	11.63	−16.23		

In the absence of significant changes in population proportions and disease patterns, the proportion of patients with different levels of complications or comorbidities should fluctuate within a stable range. However, after the implementation of the DRG payment system, there was a significant increase in the proportion of cases in certain DRG groups with higher weights and severe complications or comorbidities, such as a 26.20% increase in ET11. Conversely, there was a significant decrease in the proportion of cases in DRG groups with lower weights and without complications or comorbidities, such as a 16.23% decrease in SR15. These changes require careful attention. Subsequently, further analysis using time series analysis will be conducted to investigate whether these changes are related to the implementation of the DRG payment system.

#### 3.1.3. Analysis of the number and proportion of cases with RW <1 in each MDC before and after the implementation of the DRG payment system

DRG relative weight (RW) represents the value assigned to each DRG based on the level of resource consumption. An RW of 1 for a DRG group indicates that the medical resource consumption of that group is at the average level among all diseases, and the quantity of medical resources consumed corresponds to the severity of the disease itself. MDC stands for Major Diagnostic Category and represents a preliminary grouping step. The distribution of RW within MDC groups reflects the distribution of severity levels within each disease category.

Nineteen MDC groups with case counts > 50 were included in the analysis (see Table 4). The results show that after the implementation of the DRG payment system, there was a slight decrease in the proportion of cases with RW <1 in some MDC groups, such as a 1.86% decrease in MDCB, 4.05% decrease in MDCC, 2.53% decrease in MDCG, and 4.43% decrease in MDCI. Significant decreases were observed in some MDC groups, such as MDCE, where the proportion of cases with RW <1 decreased from 70.05% (11,661 cases) to 56.4% (6,002 cases), representing a 13.65% decrease. Similar trends were observed in MDCM and MDCS, with a decrease of 27.24 and 12.77% in the proportion of cases with RW <1, respectively.

**Table 4:** Number and proportion of cases with RW <1 in each MDC.

**MDC**	**Before implementation**		**After implementation**		**χ^2^**	***P***
	**Number of cases**	**Proportion**	**Number of cases**	**Proportion**		
MDCB	9,624	74.42	7,399	72.56	10.146	0.001
MDCC	843	63.96	505	59.91	3.603	0.058
MDCD	3,579	73.79	2,913	75.27	2.470	0.116
MDCE	11,661	70.05	6,002	56.4	529.653	<0.001
MDCF	7,935	57.16	4,751	47.91	199.136	<0.001
MDCG	8,229	76.74	5,289	74.21	14.921	<0.001
MDCH	1,639	51.01	1,532	58.05	28.934	<0.001
MDCI	4,669	77.42	3,461	72.99	28.147	<0.001
MDCJ	1,376	80.09	955	70.38	39.032	<0.001
MDCK	1,616	76.48	1,994	74.51	2.456	0.117
MDCL	2,804	56.18	2,398	58.25	3.928	0.048
MDCM	535	74.72	414	47.48	121.351	<0.001
MDCN	1,246	61.68	755	56.01	10.795	0.001
MDCO	279	75.82	179	81.00	2.143	0.143
MDCP	1,078	90.13	729	87.62	3.193	0.074
MDCQ	530	89.53	497	83.25	9.953	0.002
MDCR	8,125	94.71	6,610	92.33	36.943	<0.001
MDCS	690	69.49	755	56.72	39.387	<0.001
MDCX	575	82.38	361	64.58	51.712	<0.001

Among the 19 MDC groups, 15 groups showed a decrease in the proportion of cases with RW <1 for mild conditions. In the absence of significant changes in population proportions and disease patterns, the reasonable adjustment of the proportion of mild cases with RW <1 should be within a small fluctuation range. As shown in Table 4, among the 19 MDC groups, 16 groups had a fluctuation of <10% in the proportion of cases with RW <1. However, a more careful examination is warranted for cases with a decrease exceeding 10% or higher. The main reason for these changes is the physicians or departments' efforts to reduce the risk of overspending. Subsequently, further analysis using time series analysis will be conducted to investigate the relationship between these changes and the implementation of the DRG payment system.

#### 3.1.4. Analysis of the number and proportion of unscheduled readmission cases within 31 days in the ADRGs

Previous studies have found that after the implementation of the DRG payment system, there have been observed instances of decreased quality of diagnosis and treatment as well as decomposed hospitalizations (splitting a patient's hospital treatment for a particular disease into two separate hospitalizations with a short interval), both of which are associated with the indicator “Number and proportion of unplanned readmissions within 31 days.” Therefore, this indicator was monitored.

Out of the total of 169,778 cases in the dataset, comprising 210 ADRG groups, cases with fewer than 50 instances, planned readmissions within 31 days, cases related to cancer chemotherapy, rehabilitation, and follow-up were excluded. Subsequently, ADRG groups with unplanned readmission proportions within 0.5% (considered within the normal range) were further removed. As a result, 23 ADRG groups were identified (see Table 5). The results showed a notable increase in the proportion of unplanned readmissions within 31 days in ADRG groups such as DT1, FM3, and GZ1. Among the majority of ADRG groups listed in the table, the proportion of unplanned readmissions within 31 days increased rather than decreased. However, smaller absolute values of case counts may not be compelling enough, and thus further analysis using time series analysis will be conducted to investigate the relationship between these changes and the implementation of the DRG payment system.

**Table 5:** Number and proportion of unscheduled readmissions within 31 days in each ADRG.

**ADRG**	**Before implementation**		**After implementation**		**χ^2^**	***P***
	**Number of unscheduled readmissions within 31 days**	**Proportion**	**Number of unscheduled readmissions within 31 days**	**Proportion**		
BR2	31	0.43%	35	0.54%	0.919	0.338
BX2	12	0.59%	7	0.84%	0.539	0.463
CB1	10	2.11%	10	2.96%	0.599	0.439
DT1	9	0.56%	19	1.21%	3.771	**0.049**
ER3	7	1.40%	27	1.93%	0.598	0.439
FM3	12	0.57%	18	1.21%	4.195	**0.041**
FR3	21	0.70%	30	1.06%	2.146	0.143
FR4	10	0.72%	4	1.38%	1.288	0.257
GS1	11	2.24%	10	2.77%	0.243	0.622
GV1	18	1.83%	16	2.18%	0.265	0.607
GZ1	6	0.41%	16	1.72%	10.641	**<0.001**
HS2	13	2.57%	13	3.45%	0.576	0.448
HU1	4	1.19%	10	2.45%	1.599	0.206
KS1	6	0.46%	11	0.51%	0.040	0.841
LL1	3	1.35%	14	2.64%	1.172	0.279
LQY	18	2.76%	3	4.29%	0.524	0.469
LR1	11	1.42%	15	2.23%	1.346	0.246
LU1	9	1.19%	13	1.55%	0.381	0.537
PU1	24	2.23%	22	3.03%	1.115	0.291
QS3	8	6.78%	10	7.25%	0.021	0.884
RE1	21	10.50%	65	12.24%	0.424	0.515
SR1	7	1.86%	18	2.80%	0.890	0.346

The bold values indicate statistically significant *p*-value.

### 3.2. Intermittent time series analysis results

#### 3.2.1. Changes in the proportion of cases with severe complications or comorbidities in ADRG groups

Based on the chi-square test analysis mentioned earlier, it can be understood that in 10 ADRG groups, including ED1, ER1, ES1, ET1, FM3, FW1LV1, OS4, RW2, and SR1, the ADRG groups with subdivisions indicating severe complications or comorbidities showed an increase in case numbers after the implementation of the payment system, which was statistically significant. Additionally, among 5 ADRG groups, including ED1, ET1, FW1, OS4, and SR1, the ADRG groups with subdivisions showed significant changes in case numbers, exceeding 10%. Therefore, we used the interrupted time series method to analyze the ADRG groups with significant changes in case numbers and severe complications or comorbidities. The specific analysis results are shown in Table 6.

**Table 6:** Change in the proportion of cases in each DRG with serious comorbidities or complications before and after the implementation of the DRG payment system.

**DRG name**	**Base intercept** ***β_**0**_***		**Base slope** ***β_**1**_***		**Horizontal change** ***β_**2**_***		**Trend change** ***β_**3**_***	
	**Estimated value**	***p*** **-value**	**Estimated value**	***p*** **-value**	**Estimated value**	***p*** **-value**	**Estimated value**	***p*** **-value**
ED11	0.519	<0.0001	−0.005	0.647	−0.053	0.611	−0.010	0.622
ET11	0.623	<0.0001	0.005	0.742	−0.243	0.080	−0.023	0.388
FW11	0.530	<0.0001	−0.024	0.008	0.190	0.016	0.022	0.128
QS41	0.414	<0.0001	−0.004	0.768	−0.044	0.680	0.050	0.027
SR11	0.655	<0.0001	−0.027	0.009	0.044	0.586	0.061	0.001

From the Table 6, it can be observed that the estimated values of the baseline slope β_1_, level change β_2_, and trend change β_3_ in the ED11 subgroup are not statistically significant. The ET11 subgroup and FW11 subgroup have estimated trend change values of−0.023 and 0.022, respectively, but with *P* > 0.05, and other statistics are also not statistically significant.

**Figure 2:**
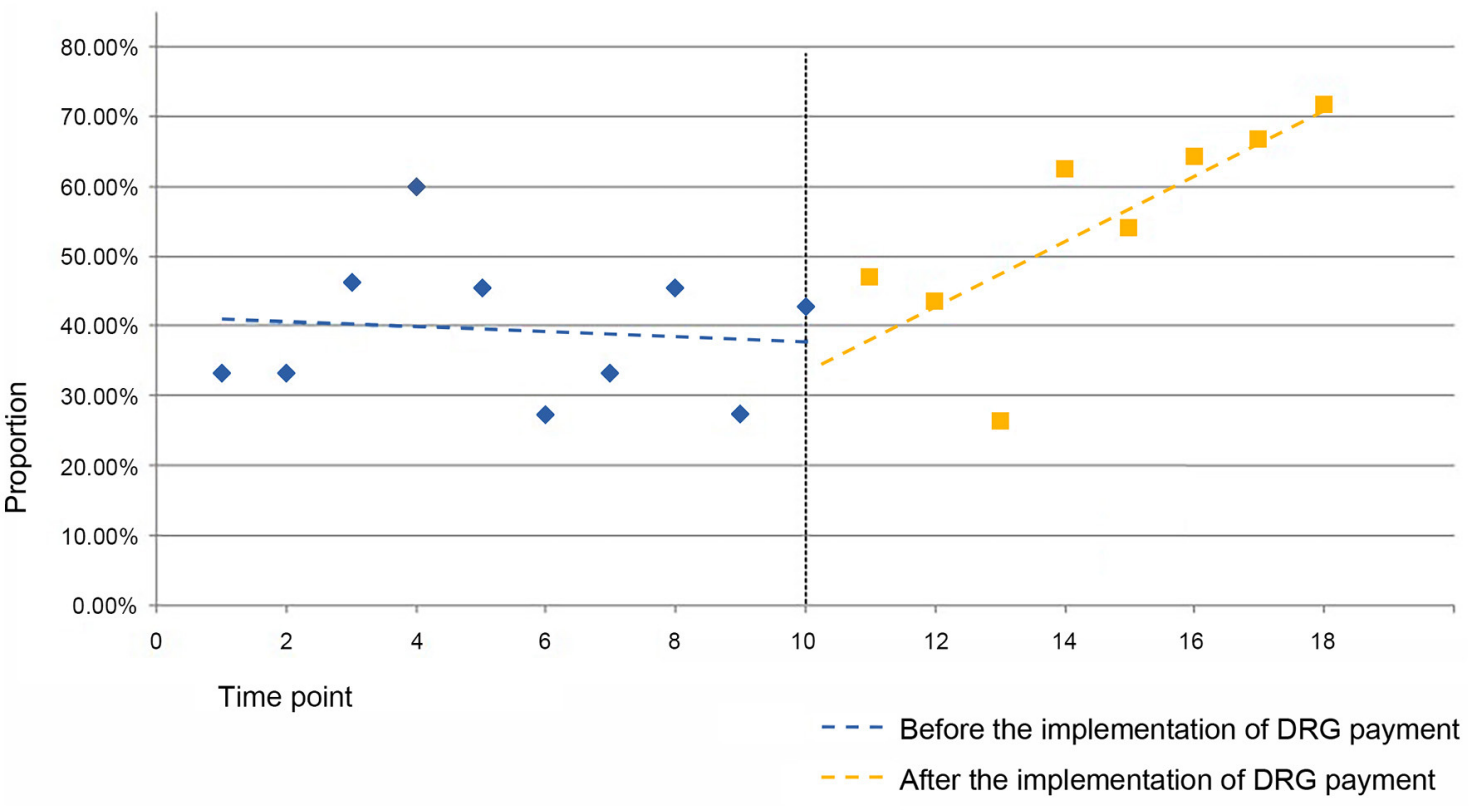
Folding line graph of the change in the proportion of cases in the QS41 group before and after the implementation of the DRG payment system.

For the QS41 group, the estimated values of the baseline slope β_1_ and level change β_2_ for the proportion of cases are not statistically significant. The estimated value of the trend change β_3_ for the proportion of cases in the QS41 group is 0.050 (*P* = 0.027 <0.05), indicating a 5-percentage-point increase in the trend change rate for the proportion of cases with severe complications or comorbidities in other anemia diseases within the QS4 group after the implementation of the DRG payment system (see Figure 2).

**Figure 3:**
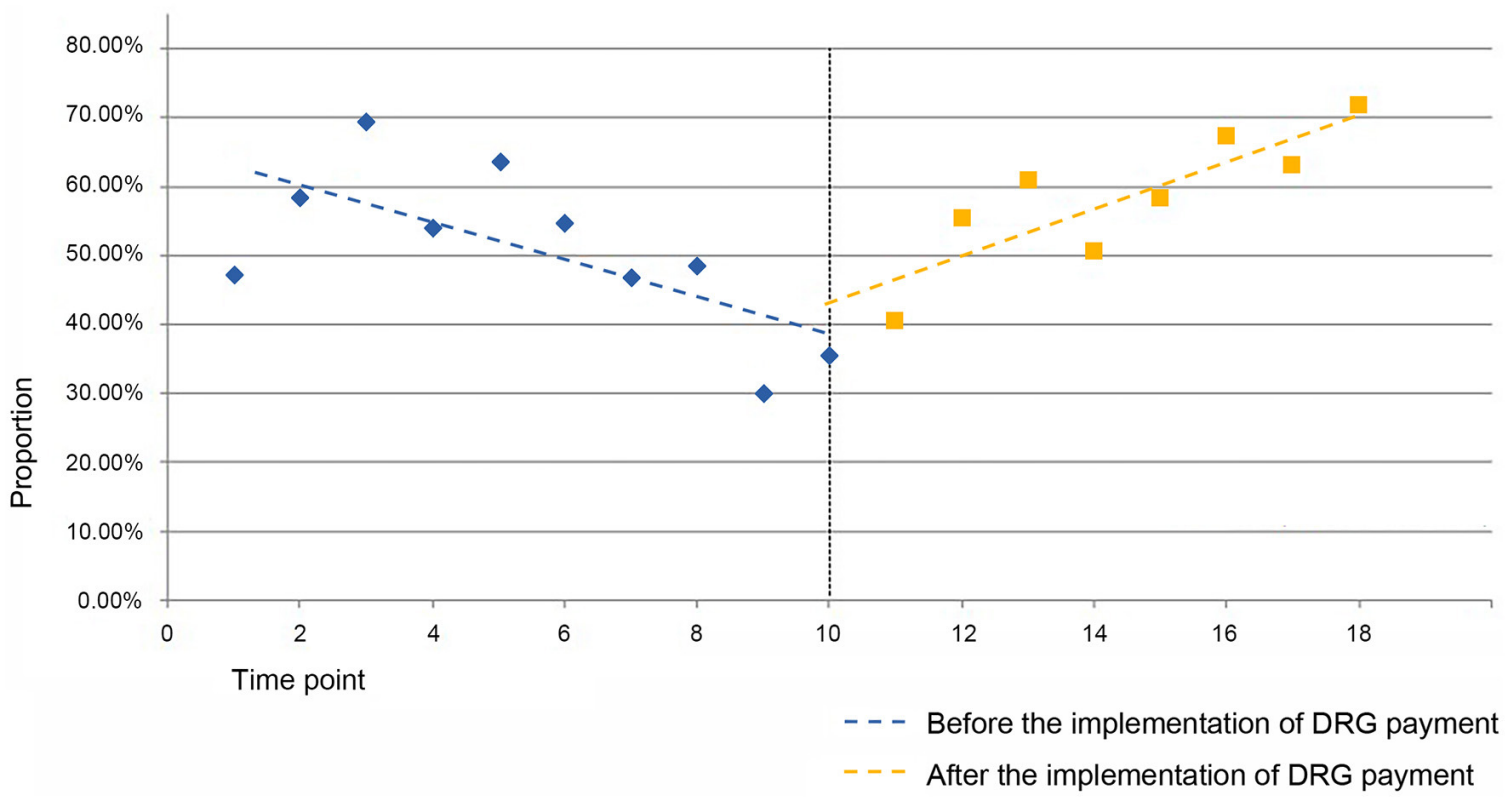
Folding line graph of the change in the proportion of cases in the SR11 group before and after DRG payment.

For the SR11 group, the estimated value of the baseline slope β_1_ for the proportion of cases (within the SR1 group) is −0.027 (*P* = 0.009 <0.05), indicating a decreasing trend in the proportion of cases within the SR11 group before the implementation of the DRG payment system, with statistical significance. The estimated value of the level change β_2_ for the proportion of cases is not statistically significant. The estimated value of the trend change β_3_ for the proportion of cases is 0.061 (*P* = 0.001 <0.05), indicating a 6.1-percentage-point increase in the trend change rate for the proportion of cases with severe complications or comorbidities in septicemia diseases within the SR1 group after the implementation of the DRG payment system (see Figure 3).

#### 3.2.2. Changes in the proportion of RW <1 cases in each MDC group

According to the chi-square test analysis mentioned earlier, significant changes were observed in the number of RW <1 cases in the MDCE, MDCM, and MDCS groups after the implementation of the DRG payment system. The time series analysis method was used to analyze the changes in the proportion of RW <1 cases in the MDCE, MDCM, and MDCS groups after the implementation of the DRG payment system. The specific analysis results are shown in Table 7.

**Table 7:** Change in the proportion of RW <1 cases in each MDC group before and after the implementation of the DRG payment system.

**MDC**	**Variable**	**Base intercept** ***β_**0**_***		**Base slope** ***β_**1**_***		**Horizontal change** ***β_**2**_***		**Trend change** ***β_**3**_***	
		**Estimated value**	***p*** **-value**	**Estimated value**	***p*** **-value**	**Estimated value**	***p*** **-value**	**Estimated value**	***p*** **-value**
MDCE	Percentage of RW <1 cases	0.665	0.000	0.005	0.241	−0.073	0.087	−0.027	0.004
MDCM	Percentage of RW <1 cases	0.737	0.000	0.004	0.843	−0.236	0.157	0.000	0.000
MDCS	Percentage of RW <1 cases	0.505	0.000	0.028	0.001	−0.022	0.716	−0.079	0.000

**Figure 4:**
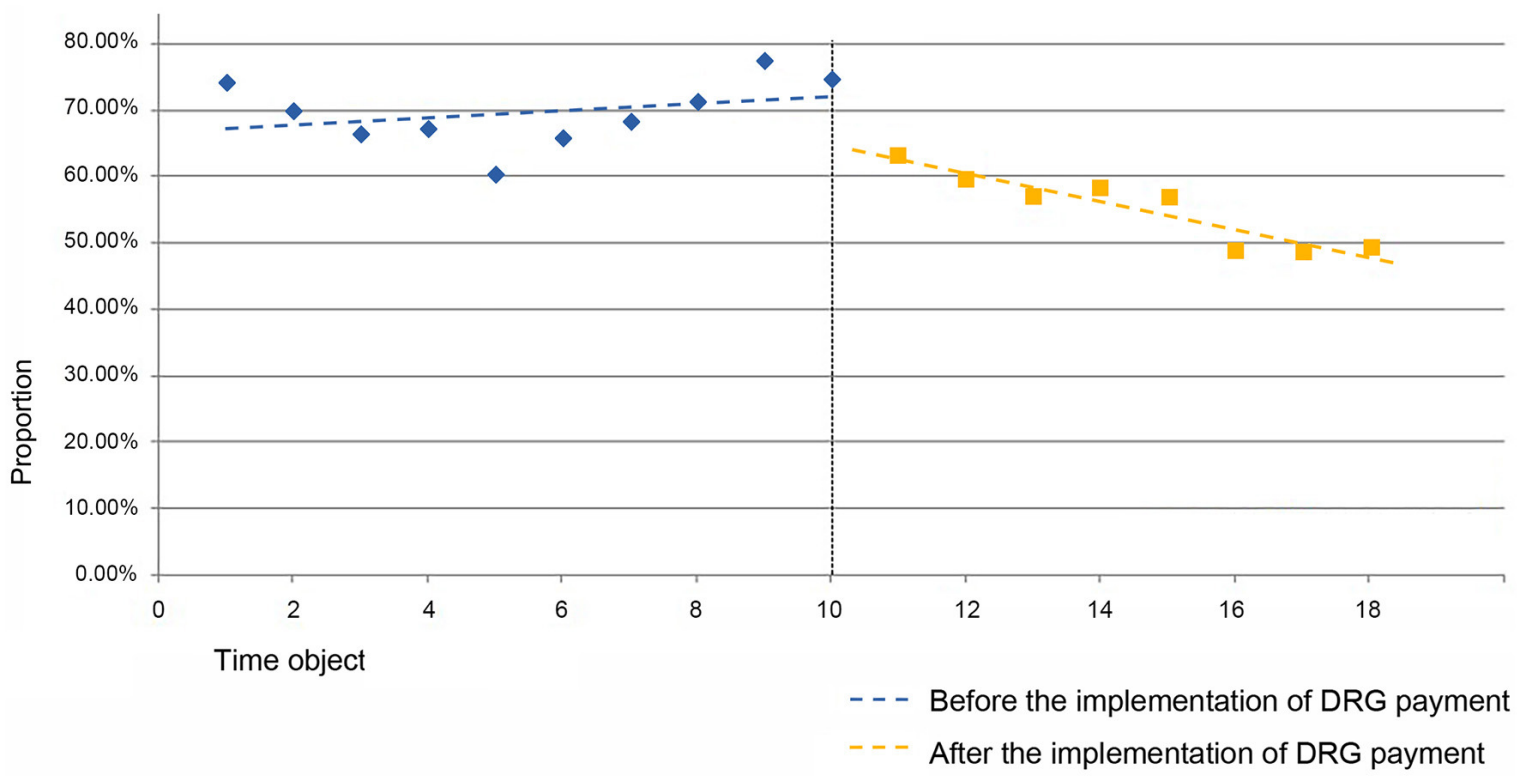
Folding line graph of the change in the proportion of cases with RW 1 in MDCE before and after the implementation of DRG payment system.

From the Table 7, it can be observed that the estimated value of the baseline slope β_1_ for the proportion of RW <1 cases in the MDCE group is 0.005 (*P* = 0.241 > 0.1), indicating an upward trend in the proportion of RW <1 cases in the MDCE group before the implementation of the DRG payment system, but without statistical significance. The estimated value of the level change β_2_ for the proportion of RW <1 cases in MDCE is −0.073 (*P* = 0.087 <0.1), indicating an instantaneous decrease of 7.3 percentage points in the proportion of RW <1 cases in MDCE after the implementation of the DRG payment system. The estimated value of the trend change β_3_ for the proportion of RW <1 cases in MDCE is−0.027 (*P* = 0.004 <0.1), indicating a decrease of 2.7 percentage points in the trend change rate for the proportion of RW <1 cases in MDCE, representing a change in the trend in the proportion of RW <1 cases of respiratory system diseases and dysfunction represented by MDCE after the implementation of the DRG payment system. The new trend change coefficient decreased by 0.027 compared to the original trend change coefficient, as shown in Figure 4.

For the MDCM group, the trend change β_3_ for the proportion of RW <1 cases has an estimated value of 0, indicating no trend change before and after the implementation of the DRG payment system.

**Figure 5:**
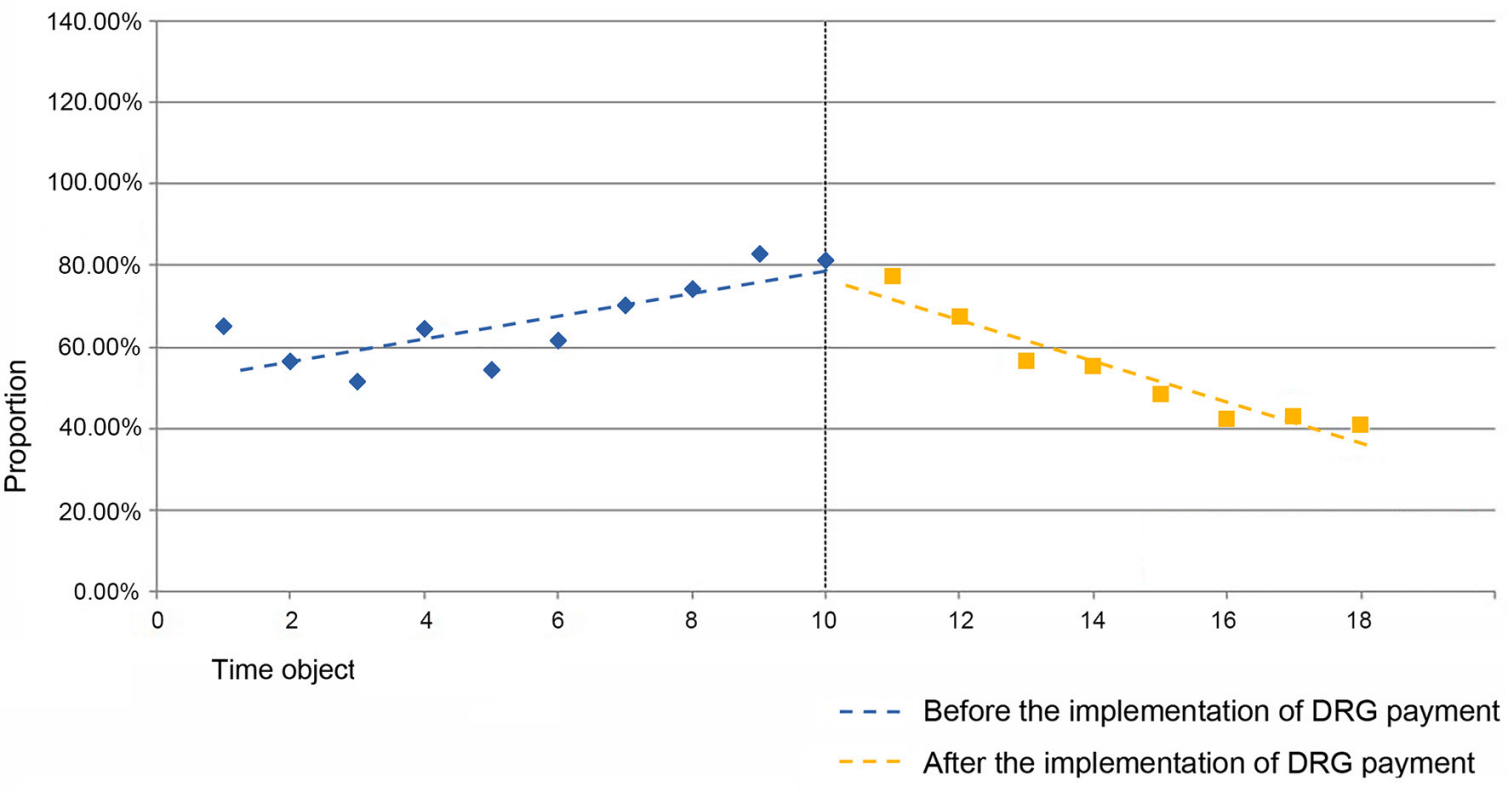
Folding line graph of the change in the proportion of cases with RW 1 in MDCS before and after the implementation of DRG payment system.

The baseline slope β_1_ for the proportion of RW <1 cases in the MDCS group has an estimated value of 2.8% per half month (*P* = 0.001 <0.1), indicating an increasing trend. At the instantaneous implementation of the DRG payment system, there is a decrease of 2.2% in the proportion of RW <1 cases, but without statistical significance. The estimated value of the trend change β_3_ for the proportion of RW <1 cases in MDCS is −0.079 (*P* = 0.000 <0.1), indicating a decrease of 7.9% points in the trend change rate for the proportion of RW <1 cases in MDCS, representing a change in the trend in the proportion of RW <1 cases of infectious and parasitic diseases (systemic or unspecified) represented by MDCS after the implementation of the DRG payment system. The new trend change rate decreased by 0.079 compared to the original 0.028 (2.8% increase per half month), resulting in −0.051 (5.1% decrease per half month), as shown in Figure 5.

#### 3.2.3. Analysis of the change in readmission rate before and after the DRG payment system reform

According to the chi-square test analysis mentioned earlier, it can be understood that ADRG groups such as DT1, FM3, and GZ1 showed significant changes in the unplanned readmission rate within 31 days before and after the implementation of the DRG payment system, and these changes were statistically significant. Using the interrupted time series method, the analysis of the unplanned readmission rate within 31 days in ADRG groups such as DT1, FM3, and GZ1 before and after the implementation of the DRG payment system was conducted. The specific analysis results are shown in Table 8.

**Table 8:** Change in the unscheduled readmission rate within 31 days in each ADRG before and after implementation of the DRG payment system.

**ADRG**	**Variable**	**Base intercept** ***β_**0**_***		**Base slope** ***β_**1**_***		**Horizontal change** ***β_**2**_***		**Trend change** ***β_**3**_***	
		**Estimated value**	***p*** **-value**	**Estimated value**	***p*** **-value**	**Estimated value**	***p*** **-value**	**Estimated value**	***p*** **-value**
DT1	Unscheduled readmission rate within 31 days	0.0034	0.000	0.0005	0.115	−0.0030	0.042	0.0015	0.035
FM3	Unscheduled readmission rate within 31 days	0.0028	0.000	0.0005	0.142	0.0027	0.072	0.0000	0.097
GZ1	Unscheduled readmission rate within 31 days	0.0063	0.000	−0.0004	0.148	0.0096	0.071	0.0013	0.065

**Figure 6:**
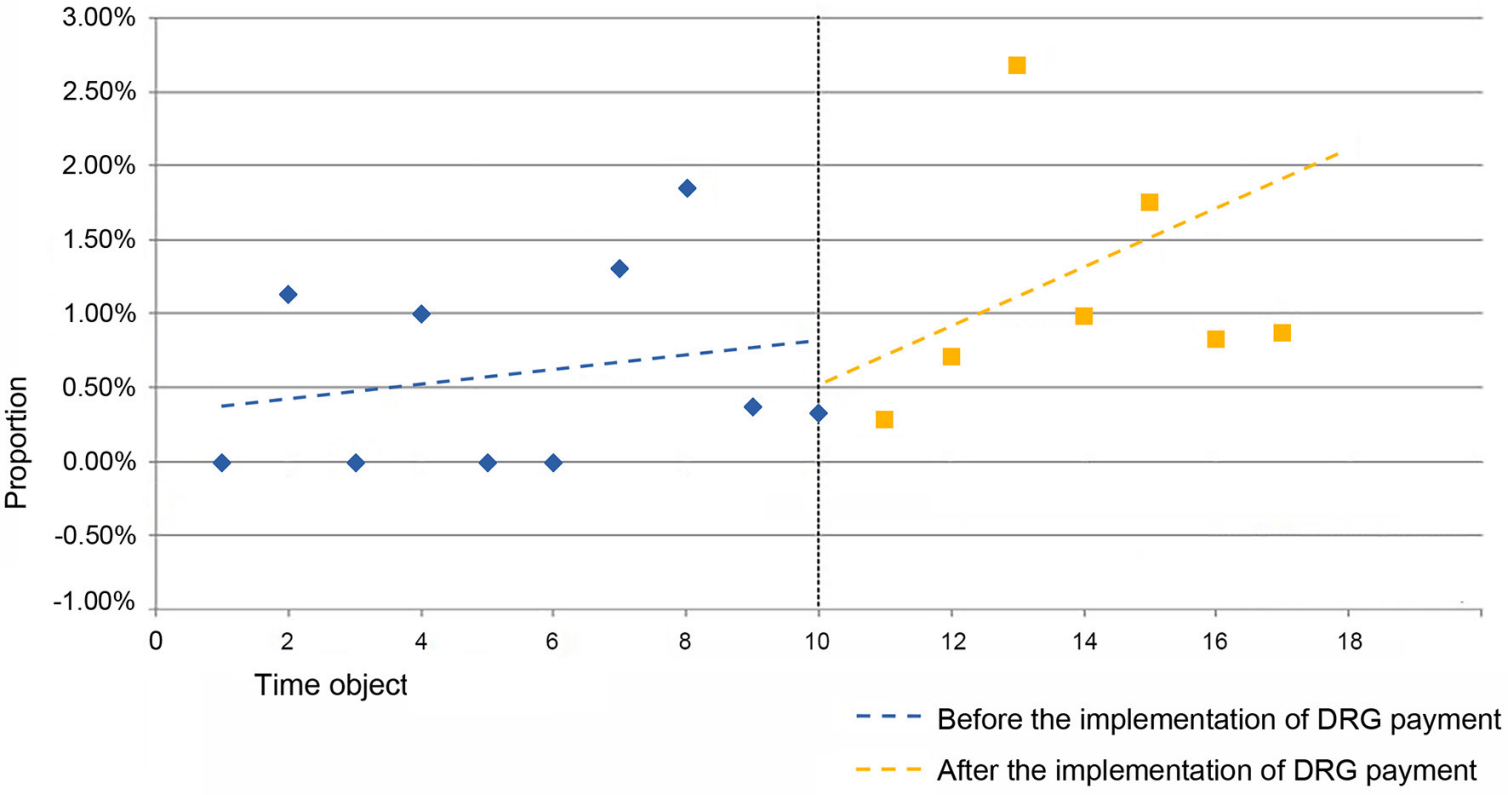
Folding line graph of the change in the unscheduled readmission rate within 31 days in the DT1 group before and after the DRG payment system.

From the Table 8, it can be observed that before the implementation of the DRG payment system, the estimated value of the baseline slope β_1_ for the unplanned readmission rate within 31 days in the DT1 group is 0.05% (*P* = 0.115 > 0.05), indicating an increasing trend in the unplanned readmission rate within 31 days in the DT1 group before the implementation of the DRG payment system, but without statistical significance. At the instantaneous implementation of the DRG payment system, there is a 0.3% decrease in the unplanned readmission rate within 31 days in the DT1 group, with a *P*-value of 0.042 <0.05, indicating statistical significance. After the implementation of the DRG payment system, the estimated value of the trend change β_3_ for the unplanned readmission rate within 31 days in DT1 is 0.0015 (*P* = 0.035 <0.05), indicating a 0.15-percentage-point increase in the trend change rate for the unplanned readmission rate within 31 days in DT1 (see Figure 6). However, for FM3 and GZ1, all the *P*-values for their baseline slope β_1_, level change β_2_, and trend change β_3_ are >0.05, indicating no statistical significance.

### 3.3. Intermittent time series analysis results

#### 3.3.1. Interview results regarding the changes in the proportion of cases with severe complications or comorbidities in DRG groups

Regarding the phenomenon of changes in the proportion of cases with severe complications or comorbidities in DRG groups, mentioned in the interrupted time series analysis, related findings and the reasons for the occurrence were mentioned during semi-structured interviews with 36 healthcare professionals. Firstly, the phenomenon of changes in the proportion of cases with severe complications or comorbidities in DRG groups was found to be widespread, often characterized by an increase in the proportion of cases with severe complications or comorbidities, and a decrease in the proportion of cases with general complications or comorbidities and those without complications or comorbidities. This phenomenon typically occurs under three circumstances: First, prior to the implementation of the DRG payment system, the reimbursement amount from medical insurance was not related to the DRG grouping but was based on the actual cost. Therefore, doctors had a more open mindset when assigning disease codes. After the implementation of the DRG payment system, disease coding became correlated with the reimbursement amount, leading to more cautious behavior among doctors. They would predict potential complications and comorbidities in the early stages of diagnosis and treatment based on their experience when assigning the fourth digit of the disease code, resulting in a higher frequency of assigning codes indicating severe or general complications or comorbidities. Second, in the Chinese healthcare system, there is a strong organizational structure within departments, and the economic interests of department members are aligned. More than 12 doctors mentioned that after the implementation of the DRG payment system, for many disease types, if the disease codes were filled based on the actual condition, the resulting medical insurance payments would be significantly lower, leading to a substantial decline in departmental revenue and even financial losses. Therefore, after internal discussions within the departments, there would be selective assignment of higher codes for certain disease types. Third, individual doctors may consciously engage in upcoding to increase their personal economic income. All 36 participating doctors acknowledged the existence of this phenomenon, but stated that it was relatively rare.

During the interviews, an internal medicine doctor stated: “Cardiovascular patients are mostly older adults and often have multiple complications/comorbidities. Although not all complications/comorbidities are identified in the early stages of diagnosis and treatment, based on experience, we tend to make early estimates. When filling in the fourth digit of the disease code, we tend to assign more codes indicating severe or general complications or comorbidities.”

#### 3.3.2. Interview results regarding the changes in the proportion of cases with RW <1 in each MDC group

MDC grouping is a classification method for major disease categories. In this study, after excluding MDC groups with fewer cases, a total of 19 MDC groups were included in the interrupted time series analysis. Most MDC groups showed a decrease in the proportion of cases with RW <1, and significant decreases were observed in the MDCE, MDCM, and MDCS groups. The interview results revealed that this phenomenon could occur under the following three circumstances: First, after the implementation of the DRG payment system, departmental revenue became the difference between medical insurance payments and treatment costs. Doctors found that the medical insurance payments for mild cases were significantly lower, leading to a substantial decrease in departmental revenue and even losses. As a result, there was a selective admission of more severe inpatients, including persuading mild patients to receive treatment at primary care hospitals or transferring their treatment from inpatient to outpatient settings. Additionally, some doctors, for their own interests, actively postponed the admission of mild patients and selectively admitted severe patients. Second, due to the same reasons mentioned earlier, to avoid a decline in departmental revenue or even losses, doctors tend to assign higher codes when a patient's disease meets the criteria for two or more disease codes, in order to receive higher reimbursement.

During the interviews, doctors mentioned the issue of an increase in the proportion of cases with RW <1 in the MDCE group (respiratory system diseases and functional disorders). They mentioned that respiratory patients in China are mostly middle-aged and older adults with relatively low socioeconomic status. Their overall health is relatively poor compared to the general patient population, and their treatment often requires more medical resources. However, the medical insurance payments cannot cover the additional resources required. Therefore, when faced with the same imaging results, doctors tend to assign higher codes for respiratory diseases. In interviews with doctors in the otorhinolaryngology department, they stated: “The development of otorhinolaryngology in China is not yet mature, and the corresponding localized DRG grouping is also not mature enough, especially in the field of ophthalmology. We prefer to treat severe patients, as it allows the department and doctors to receive higher income. Sometimes, mild patients are transferred to outpatient settings because the hospitalization costs for some mild cases are relatively high, while the medical insurance payments are lower.”

#### 3.3.3. Interview results regarding the changes in the unplanned hospitalization rate within 31 days

During the interviews, doctors from the medical administration department mentioned that the phenomenon of unplanned hospitalizations within 31 days usually occurs under two circumstances: First, it occurs due to complex diseases, severe conditions, or low treatment quality, leading to a deterioration in patients' conditions after discharge, resulting in readmission within 31 days. Second, some individual doctors engage in the practice of “decomposed hospitalization” for personal financial gain. This involves discharging patients when they are midway through their treatment, and readmitting them after a certain period to complete the remaining treatment process, thus receiving payments from medical insurance twice. However, in recent years, the practice of decomposed hospitalization has been easily detected by information systems. As a result, these doctors collaborate with doctors from other hospitals to decompose hospitalizations into two different hospitals.

#### 3.3.4. Other phenomena and behavioral changes mentioned in the interviews

In addition to the interview content related to interrupted time series analysis, the semi-structured interviews focused on behavioral changes during the implementation of the DRG payment system. The interviews also covered other phenomena and behavioral changes observed in clinical practice. Please refer to Table 9 for specific details.

**Table 9:** Other phenomena and behavioral changes mentioned in the interviews.

**Phenomenon/behavior**	**Mentioned content in the interview**
Providing services to assign cases to higher-weighted DRG groups	Manipulating the length of time patients use a ventilator to qualify for a higher-weighted DRG group, such as in the case of AH19 group.
Discharging patients before meeting the discharge criteria	Discharging patients who have not met the discharge criteria and suggesting home-based or alternative rehabilitation treatments.
Reducing the number of prescribed diagnostic tests and laboratory investigations	Omitting certain recommended diagnostic tests and examinations based on the physician's clinical judgment to save costs.
Insufficient diagnosis and treatment provided to patients	Restricting treatment options for patients with chronic diseases to control costs.
Readmitting patients after completing major diagnostic examinations	Encouraging patients to undergo major diagnostic tests in an outpatient setting before admission to manage costs.
Advising patients to purchase medications outside the hospital	Requesting patients to obtain medications outside the hospital, particularly for medications covered by the dual-channel medical insurance system.

## 4. Discussion and conclusion

### 4.1. Physicians' unexpected behaviors

Through the interrupted time series analysis, we found a significant increase in the proportion of cases with severe complications or comorbidities in some DRG groups. Combined with the findings from semi-structured interviews, we discovered that the reasons behind the increase in the proportion of cases with severe complications or comorbidities in DRG groups are not singular. The interviews indeed confirmed the existence of unexpected behaviors, such as upcoding. Doctors may adjust the diagnosis coding to assign patients to DRG groups with higher weights in order to control the risk of exceeding budgets or to achieve higher financial surplus. However, the interviews also pointed out that the main motivation for most of these behaviors is not personal gain but rather a response to the incomplete system associated with the DRG payment method. The current system does not adequately reflect the labor value of individual doctors, resulting in perceived unfairness.

Similar situations were observed in the decrease of the proportion of cases with RW <1 in various MDC groups. Doctors mentioned the existence of cherry picking behaviors, but the primary reason for such behaviors, especially during the initial implementation of the DRG payment system, is the insufficient scientific setting of weights for certain disease groups. The difference between medical insurance payments and healthcare costs does not fall within a reasonable range, forcing doctors to make unexpected choices.

Regarding the changes in the unplanned hospitalization rate within 31 days, medical administration personnel mentioned the existence of unintended behaviors related to decomposed hospitalization. However, the occurrence of such behaviors was rare, and there was no direct evidence regarding the specific motives behind these actions.

### 4.2. Qualification of unexpected behaviors

In the study, behaviors such as upcoding, cherry picking, and reduction of services were summarized as unexpected behaviors. However, the qualitative nature of unexpected behaviors has not been thoroughly discussed. In this research, we combined quantitative and qualitative approaches to deeply explore the occurrence scenarios and incentives for unexpected behaviors, as well as engage in in-depth conversations with doctors. We found that in China, where the DRG payment system was recently implemented, unexpected behaviors often occur as a result of doctors' rational choices to protect their own interests. From a policy research perspective, the implementation of these unexpected behaviors by doctors provides genuine and reasonable feedback to the DRG payment system. It represents a rational response by economic actors operating under new institutional rules. It exposes the flaws and deficiencies in the current DRG payment method and its associated system, providing authentic feedback to medical behavior supervisors and policymakers. This feedback guides them to address the exposed problems and offer appropriate solutions. Similar studies have also indicated that due to the inadequate reflection of labor value caused by the introduction of new policies, medical professionals resort to unexpected behaviors, providing the system with authentic feedback. This feedback is beneficial for the real exposure of problems and facilitates the development of solutions to bring the healthcare system back on track (22–24). Therefore, we can conclude that unexpected behaviors cannot simply be categorized as negative or positive; they are merely behavioral changes. The specific occurrence scenarios and underlying reasons need to be treated differently, and classification should be approached with caution.

### 4.3. The reasons behind physicians' unexpected behaviors in this study

An interesting finding from the interrupted time series analysis and semi-structured interviews is that, contrary to the cherry-picking phenomenon reported in previous studies where there was an increase in the proportion of cases with RW <1, the cherry-picking behavior in China is characterized by a decrease in the proportion of cases with RW <1. We have come to a discussion conclusion that the number of doctors in China is significantly lower compared to the total population, unlike in developed countries (according to WHO data, the doctor-to-population ratio in China was 23.87 per 10,000 people in 2020, while in Australia it was 54.59, Finland 43.25, France 33.24, Germany 44.59, Italy 41.26, the Netherlands 38.36, and Spain 45.77). Additionally, China has an absolute population of over 1.4 billion. Consequently, Chinese doctors have a higher level of expertise in diagnosing and treating mild diseases compared to the average level, resulting in lower average resource consumption during the treatment process. As a result, the calculated RW values for mild diseases based on historical treatment data tend to be very low. Doctors generally feel that after the implementation of the DRG payment system, treating mild patients brings them minimal personal income, leading doctors to prefer treating severe cases.

The consequences of China's large population and vast territory extend beyond this aspect. A more concerning issue is that due to the significant disparities in development levels among different regions, the CHS-DRG grouping scheme struggles to meet the diverse healthcare practice needs across China. When certain disease categories, which are underrepresented in a small number of CHS-DRG groups, occur, doctors can easily encounter difficulties in coding for DRGs. The challenges arising from excessive regional heterogeneity also manifest in the rapid advancement of medical technology in economically developed coastal provinces. The updating speed of the DRG grouping scheme fails to keep up with the pace of technological development. As one doctor mentioned, “The RW values in the DRG groups are determined based on costs from the past few years. However, with the introduction of new technologies, the corresponding treatment costs have gradually increased. As a result, to compensate for the losses caused by implementing new technologies, doctors choose to fill in additional diagnoses to place cases into DRG groups with higher weights, thus obtaining more medical insurance fund compensation.” The unique characteristics arising from the vast territory and population size present various distinct challenges, posing management and organizational challenges to hospitals, medical associations, and government departments.

The introduction section describes China's healthcare fund supervision system, which operates under the model of a large government. The National Healthcare Security Administration in China possesses significant management authority while shouldering greater social responsibilities. Unlike the separation of administrative power and judicial power in Western systems, Chinese institutional designs prioritize problem-solving and the advancement of affairs, aiming to reduce constraints and facilitate the smooth progress of healthcare payment reforms. However, this also brings inherent weaknesses in governance. The National Healthcare Security Administration, as the highest administrative unit managing healthcare funds, holds a more dominant position within the system. Consequently, it inevitably adopts more punitive measures rather than complex incentive measures, and its response to suggestions and feedback from medical institutions may be slower (25). It takes time for the practical experience gained by frontline doctors to translate into self-renewal of the system. During this period, the contradictions between the deficiencies in the DRG payment method and its associated system and the actual clinical work manifest in the form of physicians' unexpected behaviors.

## 5. Implications and conclusions

This study focused on the unexpected behaviors of physicians under the Chinese DRG payment system. Through interrupted time series analysis and semi-structured interviews, we obtained relevant results regarding the types and clinical manifestations of unexpected behaviors. As China is still in the early stage of implementing the DRG payment system, most physicians' unexpected behaviors are a normal feedback response to the imperfect system. These behaviors are beneficial for identifying the existing deficiencies in the DRG payment system and formulating corresponding measures.

The structural problems resulting from the imbalanced development of healthcare, large population size, and vast territory in China cannot be effectively resolved in the short term. However, deviations between the CHS-DRG grouping scheme and actual clinical practice (26), as well as the dominant administrative power of supervisory departments such as the National Healthcare Security Administration, can be optimized. Chinese healthcare supervisory authorities need to undergo conceptual and role transformations, engaging in negotiations, communication, and bargaining with medical institutions rather than resorting to administrative orders or unilateral decisions to handle related affairs. It is crucial to involve patient representatives as key members in negotiations, as they can act as mediators between medical insurance and healthcare, contributing to the attainment of negotiation consensus. Additionally, integrating the strength of hospital associations can enhance the overall negotiation power of hospitals. Exploring the inclusion of hospital and physician representatives (including hospital associations and medical associations) in the negotiation process for healthcare insurance payment methods and standards is necessary to achieve a win-win situation for the insured individuals, healthcare insurance funds, and the medical industry (27). An efficient feedback system is essential for improving the CHS-DRG grouping scheme. The smooth and efficient circulation of realistic information, which promptly receives feedback, enables timely resolution of issues arising from the incompatibility between the CHS-DRG grouping scheme and actual practice.

Regarding the phenomenon of actively implementing negative unexpected behaviors, it is necessary to strengthen the identification of unexpected behaviors under the DRG payment system. First, supervision techniques should be improved and regulatory costs reduced. An information-based supervision approach should be enhanced within the existing medical insurance information system by establishing an intelligent medical record verification system at the system's front end and improving the regulatory rule repository for medical record data quality. This ensures the compliance, rationality, and authenticity of medical record data. Additionally, a healthcare service behavior supervision subsystem should be established in the information system's platform for the DRG payment system. This subsystem should design key indicators for processes related to unreasonable diagnoses, irrational use of medications, unreasonable consumption of medical supplies, high positivity rates in large-scale examinations, repeated hospitalizations, and unplanned readmissions. Relevant indicator information should be made public to remind healthcare workers to avoid the occurrence of negative unexpected behaviors (28).

There are several limitations in this study. For instance, the range of the final study groups in the interrupted time series analysis could be further expanded to include cases with less noticeable data changes, or a more macroscopic study could be conducted from a big data perspective. Second, due to privacy concerns, in-depth discussions regarding the data changes in the DRG grouping were not conducted during the interviews. Therefore, further conclusions were not drawn in this regard. Third, the article did not present quantitative results for positive and negative unexpected behaviors separately. As the Chinese DRG payment system is still in its early stages, many positive and negative unexpected behaviors resulting from the inadequate measures are mixed together in the data changes. It is anticipated that in the future, as the implementation of the DRG payment system matures and stabilizes, the quantitative manifestations of different unexpected behaviors can be observed.

## Data availability statement

The data analyzed in this study is subject to the following licenses/restrictions: the data that support the findings of this study are available on request from the corresponding author. The data are not publicly available due to privacy. Requests to access these datasets should be directed to 574224075@qq.com.

## Author contributions

A-JL conceived and designed the study and was in prime charge of review and editing. X-YY and W-FC performed substantial contributions to the collection and analysis of data for the work and the writing of original draft. HL and TL interpreted the data for the work. All authors have participated in revising the work critically, have done an approval of the final version, and agreement to be accountable for the whole work in ensuring that questions related to the integrity or accuracy of any section of the work are appropriately investigated and resolved.
