# Supplementary material for: A study of the types and manifestations of physicians' unintended behaviors in the DRG payment system
Source: Front Public Health. 2023 Jun 27;11:1141981. doi: 10.3389/fpubh.2023.1141981 (PMC10333571; doi:10.3389/fpubh.2023.1141981)
Supplement: Supplementary file 1 [file Table_1.docx]

### Appendix A CHS-DRG Major Diagnostic Categories (MDCs)

| **Disease Codes** | **Disease Names** |
| --- | --- |
| MDCA | Initial Grouping of Diseases and Related Procedures |
| MDCB | Neurological Diseases and Disorders |
| MDCC | Ocular Diseases and Disorders |
| MDCD | Head, Neck, Ear, Nose, Mouth, and Pharyngeal Diseases and Disorders |
| MDCE | Respiratory System Diseases and Disorders |
| MDCF | Cardiovascular System Diseases and Disorders |
| MDCG | Digestive System Diseases and Disorders |
| MDCH | Liver, Gallbladder, and Pancreatic Diseases and Disorders |
| MDCI | Musculoskeletal Diseases and Disorders |
| MDCJ | Skin, Subcutaneous Tissue, and Breast Diseases and Disorders |
| MDCK | Endocrine, Nutritional, and Metabolic Diseases and Disorders |
| MDCL | Renal and Urinary System Diseases and Disorders |
| MDCM | Male Reproductive System Diseases and Disorders |
| MDCN | Female Reproductive System Diseases and Disorders |
| MDCO | Pregnancy, Childbirth, and Puerperium |
| MDCP | Neonatal and Other Perinatal Conditions |
| MDCO | Blood, Hematopoietic Organs, and Immune Diseases and Disorders |
| MDCR | Myeloproliferative Diseases and Disorders, Low-Grade Malignancies |
| MDCS | Infections and Parasitic Diseases (Systemic or Unspecified Site) |
| MDCT | Mental Disorders and Disorders of Mental Functions |
| MDCU | Alcohol/Drug Use and Substance-Induced Organic Mental Disorders |
| MDCV | Trauma, Poisoning, and Drug Toxicity Reactions |
| MDCW | Burns |
| MDCX | Factors Affecting Health Status and Other Medical Encounters |
| MDCY | HIV Infection Diseases and Related Procedures |
| MDCZ | Multiple Severe Trauma |
