# Supplementary material for: A study of the types and manifestations of physicians' unintended behaviors in the DRG payment system
Source: Front Public Health. 2023 Jun 27;11:1141981. doi: 10.3389/fpubh.2023.1141981 (PMC10333571; doi:10.3389/fpubh.2023.1141981)
Supplement: Supplementary file 2 [file Table_2.docx]

### Appendix B CHS-DRG Core Diagnosis Related Groups (ADRGs)

| **ADRG Codes** | **ADRG Names** |
| --- | --- |
| **MDCA** | Heart Transplantation |
| AAI | Liver Transplantation |
| AB1 | Pancreas/Kidney Combined Transplantation |
| AC1 | Pancreas Transplantation |
| AD1 | Kidney Transplantation |
| AE1 | Lung Transplantation |
| AF1 | Allogeneic Bone Marrow/Hematopoietic Stem Cell Transplantation |
| AG1 | Autologous Bone Marrow, Hematopoietic Stem Cell Transplantation |
| AG2 | Tracheostomy with Mechanical Ventilation Support for 296 Hours or ECMO |
| AH1 | Neurological Diseases and Disorders |
| **MDCB** | Craniotomy for Brain Trauma |
| BB1 | Other Craniotomy Procedures except for Trauma |
| BB2 | Intracranial Vascular Surgery with Diagnosis of Hemorrhage |
| BC1 | Ventriculoperitoneal Shunt and Revision Surgery |
| BC2 | Spinal Cord Surgery |
| BD1 | Implantation or Removal of Neurostimulator |
| BD2 | Neck and Cerebrovascular Surgery |
| BE1 | Neurovascular Interventional Treatment |
| BE2 | Other Neurosurgical Procedures |
| BJ1 | Thrombolytic Therapy for Cerebrovascular Disease |
| BL1 | Neurovascular Interventional Examination |
| BM1 | Intracranial Hemorrhagic Disorders |
| BR1 | Cerebral Ischemic Disorders |
| BR2 | Non-Traumatic Disorders of Consciousness |
| BS1 | Viral Encephalitis, Myelitis, and Meningitis |
| BT1 | Other Infections of the Nervous System |
| B12 | Neurological Tumors |
| BU1 | Neurodegenerative Disorders |
| BU2 | Demyelinating Diseases and Cerebellar Ataxia |
| BU3 | Neuropathic Pain Disorders |
| BV1 | Neurogenic Muscular Diseases |
| BV2 | Headache |
| BV3 | Congenital Disorders of the Nervous System |
| BW1 | Cerebral Palsy |
| BW2 | Cerebral Dysfunction |
| BX1 | Cranial Nerve/Peripheral Nerve Disorders" |
| BX2 | Heart Transplantation |
| BY1 | Open head injury |
| BY2 | Closed head injury |
| BY3 | Spinal cord injury and functional disorders |
| BZ1 | Other neurological disorders |
| **MDCC** | Eye diseases and functional disorders |
| CB1 | Vitreous and retinal surgery |
| CB2 | Iris surgery |
| CB3 | Lens surgery |
| CB4 | Intraocular surgery excluding retina, iris, and lens |
| CC1 | Corneal, scleral, conjunctival surgery |
| CD1 | Orbital surgery |
| CD2 | External eye surgery excluding the orbit |
| CJ1 | Other eye surgeries |
| CR1 | Malignant tumors and borderline tumors of the eye |
| CS1 | Neurovascular disorders of the eye |
| CT1 | Non-surgical treatment of anterior chamber hemorrhage and eye trauma |
| CU1 | Acute severe eye infections |
| CV1 | Various types of glaucoma |
| CW1 | Various types of cataracts |
| CX1 | Other diseases causing eye disorders |
| CZ1 | Other eye disorders |
| **MDCD** | Head, neck, ear, nose, mouth, pharynx, and functional disorders |
| DA1 | Extensive surgeries for malignant tumors of the head and neck |
| DB1 | Non-malignant extensive surgeries of the head and neck |
| DB2 | Implantation of auditory prostheses |
| DC1 | Middle ear/internal ear/lateral skull base surgery |
| DC2 | Other minor ear surgeries |
| DD1 | Rhinoplasty |
| DD2 | Nasal cavity and sinus surgery |
| DE1 | Pharynx, larynx, trachea surgery |
| DE2 | Tonsil and/or adenoidectomy |
| DG1 | Salivary gland and other salivary gland surgeries |
| DG2 | Skull/face bone surgery |
| DG3 | Lip and cleft repair surgery |
| DJ1 | Other surgeries of the head, neck, ear, nose, pharynx, and mouth |
| DK1 | Other therapeutic procedures of the head, neck, ear, nose, pharynx, and mouth |
| DR1 | Malignant tumors of the head, neck, ear, nose, pharynx, and mouth |
| DS1 | Balance disorders and hearing impairments |
| DT1 | Middle ear infection and upper respiratory tract infection |
| DT2 | Epiglottitis, laryngitis, and tracheitis |
| DU1 | Trauma and deformity of the head, neck, ear, nose, and mouth |
| DV1 | Non-malignant proliferative disorders of the head, neck, ear, nose, and throat |
| DW1 | Oral and dental-related diseases |
| DZ1 | Other Head, Neck, Ear, Nose, Throat, Pharynx, and Oral Diseases |
| **MDCE** | Respiratory System Diseases and Disorders |
| EB1 | Major Chest Surgeries |
| EC1 | Mediastinal Surgeries |
| ED1 | Other Chest Surgeries |
| EJ1 | Other Respiratory System Surgeries |
| ER1 | Respiratory System Tumors |
| ER2 | Pulmonary Embolism |
| ER3 | Pulmonary Edema and Respiratory Failure |
| ES1 | Respiratory System Tuberculosis |
| ES2 | Respiratory System Infections/Inflammation |
| ET1 | Interstitial Lung Diseases |
| ET2 | Chronic Airway Obstruction Diseases |
| EU1 | Significant Chest Trauma |
| EV1 | Respiratory System Symptoms and Signs |
| EW1 | Pleural Disorders and Pleural Effusion |
| EX1 | Asthma and Wheezing Bronchitis |
| EX2 | Pertussis and Acute Bronchitis |
| EZ1 | Other Respiratory System Disorders |
| **MDCF** | Cardiovascular System Diseases and Disorders |
| FB1 | Implantation of Cardiac Assist Systems |
| FB2 | Cardiac Valve Surgery with Cardiac Catheterization |
| FB3 | Cardiac Valve Surgery |
| FC1 | Coronary Artery Bypass Grafting with Percutaneous Transluminal Coronary Angioplasty (PTCA) |
| FC2 | Coronary Artery Bypass Grafting with Cardiac Catheterization |
| FC3 | Coronary Artery Bypass Grafting |
| FD1 | Complex Surgery for Congenital Heart Disease |
| FD2 | Routine Surgery for Congenital Heart Disease |
| FD3 | Interventional Treatment for Congenital Heart Disease |
| FE1 | Aortic Surgery |
| FF1 | Peripheral Arterial Prosthetic/Autogenous Grafting/Bypass Surgery |
| FF2 | Other Peripheral Arterial Surgeries |
| FF3 | Complex Surgery for Venous System |
| FF4 | Routine Surgery for Venous System |
| FJ1 | Other Cardiovascular System Surgeries |
| FK1 | Implantation of Permanent Pacemaker |
| FK2 | Replacement or Upgrade of Cardiac Pacemaker |
| FK3 | Implantation or Upgrade of Cardioverter Defibrillator |
| FL1 | PerCutaneous catheter ablation with Atrial Fibrillation and/or Atrial Flutter |
| FL2 | PerCutaneous catheter ablation for Arrhythmias other than Atrial Flutter or Atrial Fibrillation |
| FM1 | Percutaneous Coronary Stent Implantation |
| FM2 | Other Percutaneous Cardiovascular Treatments |
| FM3 | PerCutaneous Cardiac Catheterization Procedures |
| FN1 | Peripheral Arterial Percutaneous Endovascular Examination and/or Treatment |
| FN2 | Peripheral Venous Percutaneous Endovascular Examination and/or Treatment |
| FR1 | Heart Failure, Shock |
| FR2 | Acute Myocardial Infarction |
| FR3 | Angina Pectoris |
| FR4 | Coronary Artery Atherosclerosis/Thrombosis/Occlusion |
| FS1 | Cardiovascular System Tumors |
| FT1 | Cardiomyopathy |
| FT2 | Infective Endocarditis |
| FT3 | Valvular Diseases |
| FU1 | Severe Arrhythmias and Cardiac Arrest |
| FU2 | Arrhythmias and Conduction Disorders |
| FV1 | Congenital Heart Disease |
| FV2 | Hypertension |
| FV3 | Syncope and/or Collapse |
| FV4 | Chest Pain |
| FW1 | Arterial Diseases |
| FW2 | Venous Diseases |
| FZ1 | Other Cardiovascular System Disorders |
| **MDCG** | Digestive System Diseases and Disorders |
| GB1 | Major Surgery for Esophagus, Stomach, Duodenum |
| GB2 | Major Surgery for Small Intestine, Large Intestine (including Rectum) |
| GC1 | Other Surgeries for Esophagus, Stomach, Duodenum |
| GC2 | Other Surgeries for Small Intestine, Large Intestine (including Rectum) |
| GD1 | Appendectomy with Perforation, Abscess, Gangrene, etc. |
| GD2 | Appendectomy |
| GE1 | Groin and Abdominal Wall Surgeries |
| GE2 | Other Abdominal Surgeries |
| GF1 | Anorectal and Perianal Surgeries |
| GF2 | Other Rectal Surgeries |
| GG1 | Abdominopelvic Adhesiolysis |
| GJ1 | Other Digestive System Surgeries |
| GK1 | Other Therapeutic Endoscopic Procedures for Digestive System |
| GK2 | Gastroscopic Procedures |
| GK3 | Colonoscopic Procedures |
| GR1 | Digestive System Malignant Tumors |
| GS1 | Gastrointestinal Bleeding |
| GT1 | Inflammatory Bowel Disease |
| GU1 | Digestive Ulcers with Bleeding or Perforation |
| GU2 | Other Digestive Ulcers |
| GV1 | Digestive Tract Obstruction or Abdominal Pain |
| GW | Esophagitis, Gastritis, Enteritis |
| GZ1 | Other Digestive System Diagnoses |
| **MDCH** | Liver, Biliary, Pancreatic Diseases and Disorders |
| HB1 | Pancreatic and/or Liver Resection and/or Bypass Surgery |
| HC1 | Cholecystectomy with Common Bile Duct Surgery |
| HC2 | Common Bile Duct Surgery |
| HC3 | Cholecystectomy |
| HC4 | Other Biliary Surgeries excluding Cholecystectomy |
| HJ1 | Other Surgeries Related to Liver, Biliary, or Pancreatic Diseases |
| HK1 | Therapeutic Endoscopic Procedures for Esophageal Variceal Bleeding |
| HL1 | Diagnostic Procedures for Liver, Biliary, Pancreatic System |
| HL2 | Therapeutic Procedures for Liver, Biliary, Pancreatic System |
| HR1 | Malignant Tumors of the Liver, Biliary, Pancreatic System |
| HS1 | Liver Failure |
| HS2 | Cirrhosis of the Liver |
| HS3 | Viral Hepatitis |
| HT1 | Acute Pancreatitis |
| HU1 | Acute Biliary Disorders |
| HZ1 | Other Liver Diseases |
| HZ2 | Other Biliary Disorders |
| HZ3 | Other Pancreatic Disorders |
| **MDCI** | Musculoskeletal Diseases and Disorders |
| IB1 | Anterior-Posterior Combined Spinal Fusion |
| IB2 | Spinal Fusion Surgery |
| IB3 | Other Surgeries Related to Spine |
| IC1 | Revision/Correction Surgeries for Spinal, Shoulder, Knee, Elbow, and Ankle Prostheses |
| IC2 | Replacement Surgeries for Spinal, Shoulder, Knee, Elbow, and Ankle Joints |
| IC3 | Joint Surgeries of Spinal, Shoulder, Knee, Elbow, and Ankle excluding Replacement/Revision |
| ID1 | Minor Joint Surgeries |
| IE1 | Pelvic Marrow Excision Surgery |
| IF1 | Upper Limb Bone Surgeries |
| IF2 | Hand Surgery |
| IF3 | Femoral Surgeries |
| IF4 | Lower Limb Bone Surgeries excluding Femoral |
| IF5 | Removal/Revision of Orthopedic Fixation Devices |
| IG1 | Muscle, Tendon, Ligament Surgeries |
| IH1 | Peripheral Nerve Surgeries |
| IJ1 | Other Surgeries of the Musculoskeletal System |
| IR1 | Pelvic Fracture |
| IR2 | Femoral Neck Fracture |
| IR3 | Femoral Shaft and Distal Fractures |
| IS1 | Forearm, Wrist, Hand, or Foot Injuries |
| IS2 | Injuries excluding Forearm, Wrist, Hand, Foot |
| IT1 | Osteomyelitis |
| IT2 | Chronic Inflammatory Musculoskeletal and Connective Tissue Diseases |
| IT3 | Infectious Arthritis |
| IU1 | Bone Diseases and Other Joint Diseases |
| IU2 | Cervical, Lumbar, and Back Disorders |
| IU3 | Malignant Lesions and Pathological Fractures of Bones, Muscles, Connective Tissues |
| IV1 | Congenital Musculoskeletal Disorders excluding Spine |
| IZ1 | Rehabilitation Care for Implants/Prostheses of Musculoskeletal System |
| IZ2 | Other Disorders of Bones, Muscles, Tendons, Connective Tissues |
| **MDCJ** | Skin, Subcutaneous Tissue, and Breast Diseases and Disorders |
| JA1 | Radical Resection of Breast Malignant Tumor with Breast Reconstruction |
| JA2 | Radical Resection of Breast Malignant Tumor |
| JB1 | Breast Shaping Surgery |
| JB2 | Breast Excision Surgery |
| JB3 | Other Breast Surgeries |
| IC1 | Facial and Other Skin/Subcutaneous Tissue Shaping Procedures |
| JD1 | Skin Graft Surgery |
| JD2 | Skin Debridement Surgery |
| JJ1 | Other Surgeries of Skin, Subcutaneous Tissue |
| JR1 | Malignant Tumors of the Breast |
| JR2 | Malignant Tumors of the Skin, Subcutaneous Tissue |
| JS1 | Severe Skin Disorders |
| JS2 | Inflammatory Skin Diseases |
| JT1 | Breast, Skin, Subcutaneous Tissue Injuries |
| JU1 | Infectious Skin Diseases |
| JV1 | Non-Malignant Proliferative Lesions of the Skin, Subcutaneous Tissue |
| JV2 | Benign Lesions of the Breast |
| JZ1 | Other Skin and Breast Disorders |
| **MDCK** | Endocrine, Nutritional, Metabolic, and Disorders |
| KB1 | Adrenal Gland Surgery |
| KC1 | Pituitary Surgery |
| KD1 | Major Thyroid Surgery |
| KD2 | Parathyroid, Thyroglossal Duct, and Other Thyroid Surgeries |
| KE1 | Bariatric Surgery |
| KF1 | Skin Graft and/or Debridement for Endocrine, Nutritional, Metabolic Disorders |
| KJ1 | Other Surgeries for Endocrine, Nutritional, Metabolic Disorders |
| KR1 | Malignant Tumors of Endocrine Glands |
| KS1 | Diabetes |
| KT1 | Endocrine Disorders |
| KU1 | Nutritional Imbalances |
| KV1 | Congenital Metabolic Abnormalities |
| KZ1 | Other Metabolic Disorders |
| **MDCL** | Kidney, Urinary System Diseases and Disorders |
| LA1 | Surgery for malignant tumors of the kidney, ureter, and bladder |
| LB1 | Non-malignant surgeries of the kidney, ureter, and bladder |
| LC1 | Renal, Ureteral, and Bladder Other Surgeries |
| LD1 | Transurethral Ureteral and Bladder Surgeries |
| LE1 | Urethral Surgery |
| LF1 | Placement, Adjustment, Removal of Renal Assist Device |
| LJ1 | Other Surgeries of the Urinary System |
| LK1 | Urinary Tract Stone Lithotripsy |
| LL1 | Renal Dialysis |
| LR1 | Renal Insufficiency |
| LS1 | Nephritis and Renal Diseases |
| LT1 | Renal and Urinary Tract Tumors |
| LU1 | Renal and Urinary Tract Infections |
| LV1 | Hypertensive/Diabetic Nephropathy |
| LW1 | Renal and Urinary Tract Signs and Symptoms |
| LX1 | Urinary Tract Stones, Obstructions, and Urethral Stricture |
| LY1 | Renal and Urinary Tract Injuries |
| LZ1 | Other Disorders of the Kidney and Urinary System |
| **MDCM** | Male Reproductive System Diseases and Disorders |
| MA1 | Male Reproductive Organ Malignant Tumor Surgery |
| MB1 | Prostate Surgery |
| MC1 | Penile Surgery |
| MD1 | Testicular Surgery |
| MJ1 | Other Surgeries of the Male Reproductive System |
| MR1 | Male Reproductive System Malignant Tumors |
| MS1 | Male Reproductive System Inflammation |
| **MDCN** | Other Disorders of the Male Reproductive System |
| NA1 | Female Reproductive System Diseases and Disorders |
| NA2 | Extensive Resection Surgery for Malignant Tumors of Female Reproductive Organs |
| NB1 | Non-Extensive Resection Surgeries for Malignant Tumors of Female Reproductive Organs |
| NC1 | Reconstructive Surgeries of the Female Reproductive System |
| ND1 | Uterine (excluding Endometrium) Surgeries |
| NE1 | Adnexal Surgeries |
| NF1 | Endometrial Surgeries |
| NG1 | Vulvar, Vaginal, Cervical Surgeries |
| NJ1 | Assisted Reproductive Technology |
| NR1 | Other Surgeries of the Female Reproductive System |
| NS1 | Malignant Tumors of the Female Reproductive System |
| NZ1 | Infections of the Female Reproductive System |
| **MDCO** | Other Disorders of the Female Reproductive System |
| OB1 | Pregnancy, Childbirth, and Puerperium |
| OC1 | Cesarean Section |
| OD1 | Vaginal Delivery with Operative Procedures |
| OD2 | Uterine and Adnexal Surgeries Related to Pregnancy |
| OE1 | Ectopic pregnancy surgery |
| OF1 | Mid-trimester induction of labor procedure |
| OF2 | Early abortion procedure |
| OJ1 | Other surgeries related to pregnancy and delivery |
| OR1 | Vaginal delivery |
| OS1 | Postpartum-related conditions |
| OS2 | Complications related to abortion |
| OT1 | Ectopic pregnancy |
| OZ1 | Other conditions related to pregnancy |
| **MDCP** | Newborn and other perinatal conditions |
| PB1 | Newborn (<29 days old) cardiovascular surgery |
| PC1 | Newborn (<29 days old) abdominal surgery |
| PJ1 | Other surgeries for newborns (<29 days old) |
| PK1 | Newborn with respiratory support |
| PR1 | Neonatal respiratory distress syndrome |
| PS1 | Extreme immaturity (birth weight <1500g) |
| PT1 | Premature infant (birth weight 1500-2499g) |
| PT2 | Premature infant (birth weight >2499g) |
| PU1 | Full-term infant |
| PV1 | Infant conditions diagnosed in infants aged 29 days to 1 year |
| **MDCQ** | Blood, hematopoietic organs, and immune disorders |
| QB1 | Splenic surgery |
| QC1 | Thymus surgery |
| QD1 | Other surgeries of the blood, hematopoietic organs, and immune system |
| QJ1 | Benign tumors in unspecified sites, tissues, or organs |
| QR1 | Vascular endothelial and immune disorders |
| QS1 | Red blood cell disorders and nutritional anemias |
| QS2 | Hemolytic anemia, aplastic anemia |
| QS3 | Other types of anemia |
| QS4 | Coagulation disorders |
| QT1 | Hemolytic anemia, aplastic anemia |
| **MDCR** | Myeloproliferative disorders and dysplastic tumors |
| RA1 | Lymphoma, leukemia, and other malignancies requiring major surgery |
| RA2 | Lymphoma, leukemia, and other malignancies requiring other surgeries |
| RA3 | Myeloproliferative disorders or dysplastic tumors requiring major surgery |
| RA4 | Myeloproliferative disorders or dysplastic tumors requiring other surgeries |
| RB1 | Chemotherapy and/or other treatments for acute leukemia |
| RC1 | Radiation therapy for malignant proliferative disorders |
| RD1 | Interventional and/or radiofrequency therapy for malignant proliferative disorders |
| RE1 | Chemotherapy and/or targeted/biological therapy for malignant proliferative disorders |
| RF1 | Terminal stage treatment for malignant proliferative disorders |
| RR1 | Acute leukemia |
| RS1 | Lymphoma and other types of leukemia |
| RS2 | Multiple myeloma |
| RT1 | Unspecified malignant tumors |
| RT2 | Unspecified benign tumors |
| RU1 | Malignant proliferative disorders associated with chemotherapy and/or targeted/biological therapy |
| RU2 | Immunotherapy for malignant proliferative disorders |
| RV1 | Malignant proliferative disorders associated with radiation therapy |
| RW1 | Follow-up examinations after treatment of malignant proliferative disorders |
| RW2 | Maintenance therapy for malignant proliferative disorders |
| **MDCS** | Infections and parasitic diseases (systemic or unspecified site) |
| SB1 | Surgical management of systemic infections |
| SR1 | Sepsis |
| SS1 | Infections after surgery and trauma |
| ST1 | Fever of unknown origin |
| SU1 | Viral diseases |
| SV1 | Bacterial diseases |
| SZ1 | Other infectious or parasitic diseases |
| **MDCT** | Mental disorders and functional impairments |
| TB1 | Surgery in patients with mental illness |
| TR1 | Schizophrenia |
| TR2 | Paranoia and acute psychosis |
| TS1 | Severe mood disorders |
| TS2 | Neurotic disorders and other affective disorders |
| TT1 | Eating and sleep disorders |
| TT2 | Personality disorders |
| TU1 | Childhood mental developmental disorders |
| TV1 | Anxiety disorders |
| TW1 | Organic and symptomatic mental disorders |
| **MDCU** | Alcohol/drug use and substance-induced organic mental disorders |
| UR1 | Alcohol intoxication and withdrawal |
| US1 | Stimulant abuse and dependence |
| **MDCV** | Trauma, poisoning, and drug toxicity reactions |
| VB1 | Skin grafting for injuries |
| VC1 | Debridement surgery for injury-related wounds |
| VR1 | Other surgeries for injuries |
| VJ1 | Injuries |
| VS1 | Allergic reactions |
| VS2 | Drug poisoning or toxic reactions |
| VT1 | Medical sequelae |
| VZ1 | Other disorders related to injuries, poisoning, and toxic reactions |
| **MDCW** | Burns |
| WB1 | Skin grafting for burns involving more than 30% body surface area or multiple third-degree burns |
| WC2 | Other skin grafting for burns |
| WJ1 | Surgical procedures for burns other than skin grafting |
| WR1 | Burns involving more than 30% of body surface area or multiple third-degree burns, corrosive burns, and frostbite |
| WZ1 | Other burns, corrosive burns, and frostbite |
| **MDCX** | Factors affecting health and other medical conditions |
| XJ1 | Other diagnostic procedures with operating room involvement |
| XR1 | Rehabilitation |
| XR2 | Other rehabilitation therapies |
| XS1 | Signs and symptoms |
| XS2 | Follow-up care (excluding malignant neoplasms) |
| XT1 | Other post-care |
| XT2 | Nonspecific congenital anomalies |
| XT3 | Other factors affecting health status |
| **MDCY** | HIV infection and related procedures |
| YC1 | Operating room surgeries for HIV-related conditions |
| YR1 | HIV-related conditions |
| YR2 | Other HIV-related situations |
| **MDCZ** | Multiple severe injuries |
| ZB1 | Craniotomy for multiple severe injuries |
| ZC1 | Spinal, cord, hip, or limb surgeries for multiple severe injuries |
| ZD1 | Abdominal surgeries for multiple significant injuries |
| ZJ1 | Other operating room procedures related to multiple significant injuries |
| ZZ1 | Multiple significant injuries without surgery |
